# Structural analysis of extracellular ATP-independent chaperones of streptococcal species and protein substrate interactions

**DOI:** 10.1128/msphere.01078-24

**Published:** 2025-01-29

**Authors:** Charles Agbavor, Madeline Torres, Nicole L. Inniss, Sarah Latimer, George Minasov, Ludmilla Shuvalova, Zdzislaw Wawrzak, Dominika Borek, Zbyszek Otwinowski, Peter J. Stogios, Alexei Savchenko, Wayne F. Anderson, Karla J. F. Satchell, Laty A. Cahoon

**Affiliations:** 1 Department of Biological Sciences, University of Pittsburgh, Pittsburgh, Pennsylvania, USA; 2 Department of Microbiology and Immunology, Northwestern University, Feinberg School of Medicine, Chicago, Illinois, USA; 3 Center for Structural Biology of Infectious Diseases, Northwestern University, Feinberg School of Medicine, Chicago, Illinois, USA; 4 Department of Pharmacology, Northwestern University, Feinberg School of Medicine, Chicago, Illinois, USA; 5 Northwestern Synchrotron Research Center, Life Sciences Collaborative Access Team, Northwestern University, Argonne, Illinois, USA; 6 Department of Biophysics, University of Texas Southwestern Medical Center, Dallas, Texas, USA; 7 Department of Biochemistry, University of Texas Southwestern Medical Center, Dallas, Texas, USA; 8 Biozone, Department of Chemical Engineering and Applied Chemistry, University of Toronto, Toronto, Ontario, Canada; 9 Department of Microbiology, Immunology, and Infectious Diseases, University of Calgary, Calgary, Alberta, Canada; 10 Department of Biochemistry and Molecular Genetics, Northwestern University, Feinberg School of Medicine, Chicago, Illinois, USA; The University of Iowa, Iowa City, Iowa, USA

**Keywords:** *Streptococcus*, ATP-independent chaperone, PPIase, PrsA, SlrA, PpiA, chaperone, secretion, Ply, pneumolysin, X-ray crystallography, pore forming toxin

## Abstract

During infection, bacterial pathogens rely on secreted virulence factors to manipulate the host cell. However, in gram-positive bacteria, the molecular mechanisms underlying the folding and activity of these virulence factors after membrane translocation are not clear. Here, we solved the protein structures of two secreted parvulin and two secreted cyclophilin-like peptidyl-prolyl isomerase (PPIase) ATP-independent chaperones found in gram-positive streptococcal species. The extracellular parvulin-type PPIase, PrsA in *Streptococcus pneumoniae* and *Streptococcus mutans* maintain dimeric crystal structures reminiscent of folding catalysts that consist of two domains, a PPIase and foldase domain. Structural comparison of the two cyclophilin-like extracellular chaperones from *S. pneumoniae* and *Streptococcus pyogenes* with other cyclophilins demonstrates that this group of cyclophilin-like chaperones has novel structural appendages formed by 9- and 24-residue insertions. Furthermore, we demonstrate that deletion of *prsA* and *slrA* genes impairs the secretion of the cholesterol-dependent pore-forming toxin, pneumolysin in *S. pneumoniae*. Using protein pull-down and biophysical assays, we demonstrate a direct interaction between PrsA and SlrA with Ply. Then, we developed chaperone-assisted folding assays that show that the *S. pneumoniae* PrsA and SlrA extracellular chaperones accelerate pneumolysin folding. In addition, we demonstrate that SlrA and, for the first time, *S. pyogene*s PpiA exhibit PPIase activity and can bind the immunosuppressive drug, cyclosporine A. Altogether, these findings suggest a mechanistic role for streptococcal PPIase chaperones in the activity and folding of secreted virulence factors such as pneumolysin.

**IMPORTANCE:**

Streptococcal species are a leading cause of lower respiratory infections that annually affect millions of people worldwide. During infection, streptococcal species secrete a medley of virulence factors that allow the bacteria to colonize and translocate to deeper tissues. In many gram-positive bacteria, virulence factors are secreted from the cytosol across the bacterial membrane in an unfolded state. The bacterial membrane-cell wall interface is exposed to the potentially harsh extracellular environment, making it difficult for native virulence factors to fold before being released into the host. ATP-independent PPIase-type chaperones, PrsA and SlrA, are thought to facilitate folding and stabilization of several unfolded proteins to promote the colonization and spread of streptococci. Here, we present crystal structures of the molecular chaperones of PrsA and SlrA homologs from streptococcal species. We provide evidence that the *Streptococcus pyogenes* SlrA homolog, PpiA, has PPIase activity and binds to cyclosporine A. In addition, we show that *Streptococcus pneumoniae* PrsA and SlrA directly interact and fold the cholesterol-dependent pore-forming toxin and critical virulence determinant, pneumolysin.

## INTRODUCTION

Molecular chaperones are essential for protein folding and the prevention of protein aggregation within the cell (1). While some chaperones are ATP-dependent, others, such as the gram-positive PrsA and SlrA, are ATP-independent (2–4). To colonize and cause disease, gram-positive bacterial pathogens depend on ATP-independent surface-exposed extracellular chaperones for full activity of secreted virulence factors (5). Secreted factors are necessary for extracellular signal sensing, host-cell attachment and host invasion (6, 7). However, in gram-positive bacteria, how secreted factors are folded, then targeted to the cell wall, or released into the host after bacterial membrane translocation is still not clear.

The *Streptococcus* genus is composed of at least 100 species of gram-positive bacteria that are found in humans and other animals (8). Although most streptococci are commensal organisms, some species such as *Streptococcus pneumoniae* (the pneumococcus), *Streptococcus pyogenes,* and *Streptococcus mutans* can cause fatal human infections, such as pneumonia, meningitis, necrotizing fasciitis, and sepsis (8, 9). Virtually all clinical isolates of pathogenic streptococci encode ATP-independent extracellular chaperones that localize to the bacterial surface. Indeed, it is hypothesized that the ATP-independent chaperones are critical for the activity of secreted protein factors; however, the molecular mechanisms underlying secreted protein folding and activity post-membrane translocation is largely unknown in gram-positive bacteria. Although ATP-independent chaperones have common features, their subcellular localization is varied and they have a broad range of substrates that are not all conserved (10, 11). In *S. pneumoniae*, we recently determined that the pore-forming toxin pneumolysin (Ply) is altered in secreted fractions when the extracellular chaperones PrsA and SlrA are absent (12), suggesting that these chaperones are important for Ply maturation.

*S. pneumoniae* PrsA and SlrA are ATP-independent chaperones that belong to the superfamily of peptidyl-prolyl isomerases (PPIases) that catalyze the *cis*-to-*trans* isomerization of residues N-terminal to proline and are ubiquitously expressed in prokaryotes and eukaryotes (13–19). Two types of ATP-independent extracellular PPIases exist in pathogenic streptococci: the parvulin and cyclophilin families. Unlike cytoplasmic PPIases, extracellular PPIases have canonical secretion signal peptides that allow for their translocation across the cell membrane via the Sec pathway (19–21). These chaperones are lipidated to the outer leaflet of the membrane and function in the space between the bacterial membrane and cell wall (19). *S. pneumoniae* PrsA (*Spn*PrsA) is critical for colonization and invasive disease (12, 22, 23) and is conserved in *S. pneumoniae* serotypes despite lacking key residues important for PPIase activity (15, 23, 24). In contrast, *S. pneumoniae* SlrA (*Spn*SlrA) is a functional PPIase and, like *Spn*PrsA, is also critical for the colonization of the upper airways as well as for invasive disease (12, 15). Other streptococcal-secreted PPIases, such as *S. mutans* PrsA (*Smu*PrsA), are required for cell wall and membrane integrity (20) and virulence in an infective endocarditis model (25). Additionally, the secretion of mutacin, the major factor in the colonization and establishment of *S. mutans* dental biofilms, is dependent on *Smu*PrsA (26). Despite the importance of streptococcal extracellular PPIases in the regulation of secreted factors, the direct interaction with specific substrate proteins remains to be shown.

In *S. pneumoniae*, the cholesterol-dependent cytolysin (CDC) Ply is important for disease causation as *ply* deletion mutants demonstrate a significant decrease in virulence in murine models (27, 28). Ply was shown to be secreted and localized to the cell wall in 18 different serotypes of *S. pneumoniae* (29). Additionally, Ply is thought to be secreted from the cytosol in an unfolded state and must rapidly fold between the membrane and cell wall to become functional (29, 30). Interestingly, in the distantly related gram-positive bacteria *Listeria monocytogenes*, *prsA2* deletion mutants demonstrate decreased secreted listeriolysin O (Ply homolog, 44% sequence identity) activity, suggesting that a PPIase chaperone is required for full CDC function (24, 31, 32). Therefore, we hypothesized that in *S. pneumoniae*, Ply may require PrsA foldase and/or SlrA PPIase activity.

Here, we determined X-ray structures of *S. pneumoniae* PrsA and SlrA, *Smu*PrsA, and *S. pyogenes* PpiA (*Spy*PpiA). The structures of *Spn*SlrA and *Spy*PpiA are the first gram-positive cyclophilin crystal structures, and for the first time, we demonstrate that *Spy*PpiA, like *Spn*SlrA, exhibits PPIase activity *in vitro* and binds cyclosporine A. Furthermore, we find that *S. pneumoniae prsA* and *slrA* deletion mutants have reduced functional Ply activity. Then using protein pull-down, biophysical, and chaperone-assisted folding assays, we demonstrate that both *S. pneumoniae* PrsA and SlrA directly interact with and accelerate the folding of Ply. Taken together, our results suggest that Ply is a substrate of the *S. pneumoniae* secretion chaperones PrsA and SlrA.

## RESULTS

### Structural comparison of streptococcal PrsA to other parvulins

As key streptococcal virulence factors are dependent on PrsA for activity (12, 22), we began characterizing streptococcal PrsA by determining the X-ray crystal structures of PrsA from *S. pneumoniae* and *S. mutans*. The *Spn*PrsA and *Smu*PrsA structures were solved to a resolution of 2.55 Å and 3.15 Å, respectively. The quality and refinement statistics for both structures are listed in Table 1. The final model of *Spn*PrsA (PDB 5TVL) contains polypeptide chains A–D, four glycerol molecules, and four chloride and two sulfate ions in the asymmetric unit (Fig. S1). Dimers 1 (chains A and D) and 2 (chains B and C) establish a dimer of dimers. Protomers of dimer 1 contain residues 27–299 and of dimer 2 contain residues 28–299 of the *S. pneumoniae* PrsA primary sequence. Dimers 1 and 2 interact in the crystal via a 1,971 Å interface (33) formed primarily between cognate foldase (N) domains and a single PPIase domain (Fig. S1).

**TABLE 1:** Data quality and refinement statistics^*a*^

Statistic	Value for structure with PDB accession code:	
	5TVL	7L75
Data collection		
Space group	*P2_1_*	*C2*
Unit cell parameters (Å; °)	*a* = 72.32, *b* = 70.94, *c* = 116.63; α = 90.00, β = 91.49, γ = 90.00	*a* = 133.79, *b* = 66.21, *c* = 90.25; α = 90.00, β = 113.11, γ = 90.00
Resolution range (Å)	50.00–2.45 (2.49–2.45)	30.00–3.15 (3.20–3.15)
No. of reflections	43,458 (2,008)	12,763 (621)
*R*_merge_ (%)	9.0 (100.0)	5.6 (80.1)
Completeness (%)	98.7 (90.9)	100.0 (100.0)
⟨*I*/*σ*(*I*)⟩	20.0 (2.3)	29.0 (2.6)
Multiplicity	9.8 (6.2)	5.0 (5.1)
Wilson *B* factor	37.1	127.3
Refinement		
Resolution range (Å)	50.00–2.55 (2.62–2.55)	29.46–3.15 (3.23–3.15)
Completeness (%)	82.4 (38.2)	99.8 (100.0)
No. of reflections	30,311 (1,088)	12,118 (933)
*R*_work_/*R*_free_, (%)	22.5/29.7 (32.2/39.7)	26.6/31.0 (37.6/37.7)
Protein chains/atoms	4/8,452	2/4,198
Ligand/Solvent atoms	64/91	36/0
Mean temperature factor (Å^2^)	56.4	155.3
Coordinate deviations		
R.m.s.d. bonds (Å)	0.007	0.002
R.m.s.d. angles (°)	0.797	1.100
Ramachandran plot		
Favored (%)	98.0	87.0
Allowed (%)	2.0	13.0
Outside allowed (%)	0.0	0.0

^*a*^ Values in parentheses are for the outer shell. R.m.s.d., root-mean-square deviation.

The final model of *Smu*PrsA (PDB 7L75) contains only two polypeptide chains, A and B, comprised of residues 28–294 and 29–294, respectively. The root-mean-square deviation (r.m.s.d.) between chains A and B is 0.70 Å. *Smu*PrsA and *Spn*PrsA (dimer 1) align well with an r.m.s.d. of 3.76 Å across all Cα atoms (Fig. S2A). As there is flexibility between the domains of PrsA, we generated a flexible pairwise alignment of a single polypeptide chain from each structure using the FATCAT server (34). As expected, *Spn*PrsA and *Smu*PrsA align well with an r.m.s.d. of 2.36 Å across 264 Cα atoms that share 49% sequence identity (Fig. S2B).

**Fig 1:**
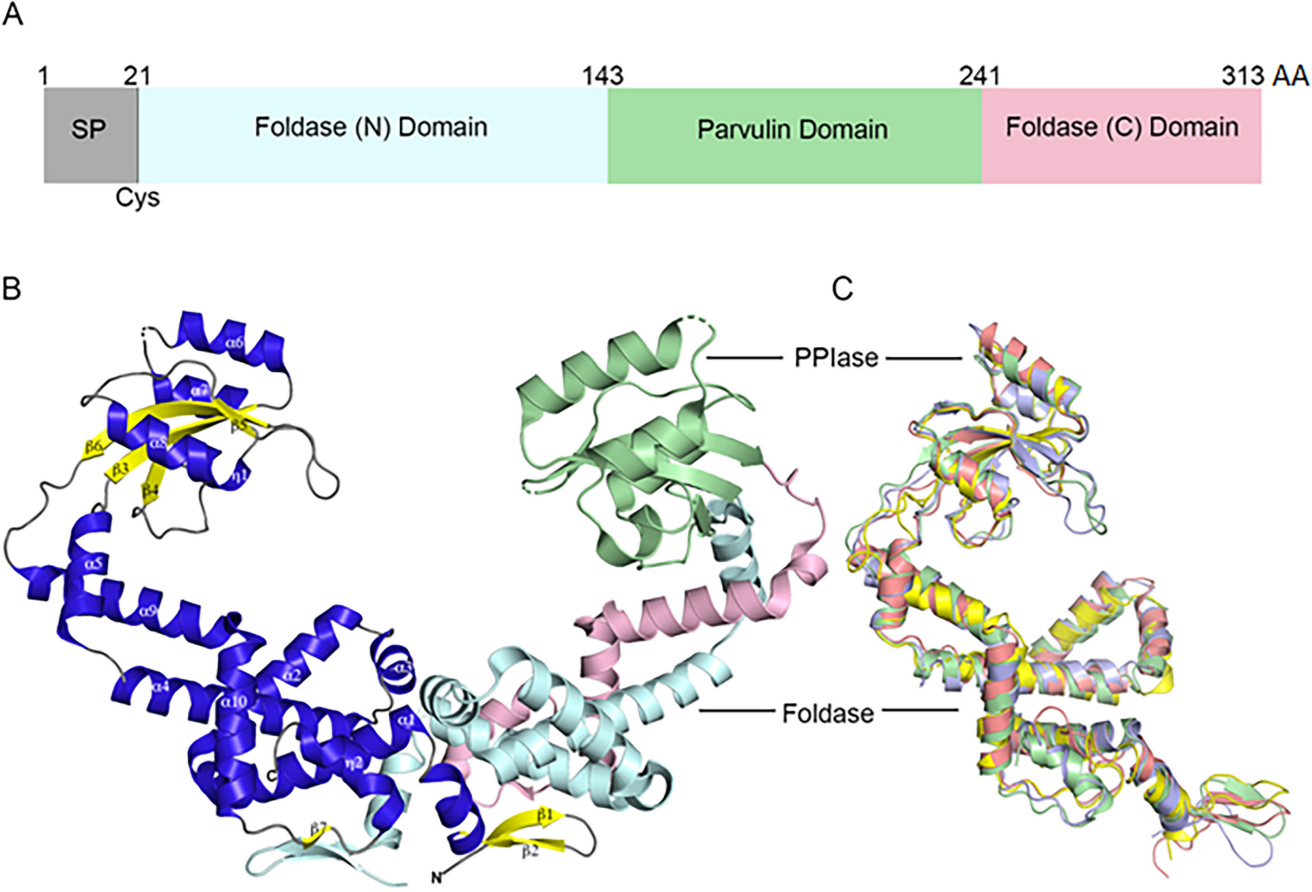
Overview of streptococcal PrsA structure. (**A**) The domain organization of the *S. pneumoniae* (*Spn*PrsA) protomer derived with the InterProscan (36). Amino acids 1–20 encode the signal peptide (SP) and amino acid 21 is the conserved lipid-modified cysteine (Cys) residue (37), 22–142 encode the foldase domain N-terminal region (light cyan), 143–241 encode the parvulin domain (pale green), residues 242–313 encode the foldase domain C-terminal region (pale pink). (**B**) Cartoon representation of the overall dimeric structure of *Spn*PrsA (PDB 5TVL). The left protomer is colored with α-helices in blue, β-strands in yellow, and loop regions in gray. The α-helices and β-strands are labeled according to secondary structure elements as detailed previously (13), as are the N and C termini. The right protomer is colored by domain as in panel A. (**C**) A flexible overlay of the structures of *S. pneumoniae* (PDB 5TVL, pale green), *S. mutans* (PDB 7L75, violet), *Bacillus subtilis* (PDB 4WO7, yellow), and *L. monocytogenes* (PDB 5HTF, salmon) PrsA proteins.

Consistent with PrsA homologs, both streptococcal PrsA proteins have a central parvulin PPIase domain (*Spn*PrsA residues 143–241) linked to a foldase domain (N- and C-terminal regions) (Fig. 1A and B) (13, 35). The PPIase domain is composed of a four-stranded antiparallel β-sheet (β3, 146–153; β4, 192–195; β5, 222–224; β6, 233–242) framed by four helices (α6, 155–168; α7, 172–181; α8, 203–213; η1, 183–188). The remaining residues comprise the foldase domain which contains eight helices (α1, 40–71; α2, 76–91; α3, 93–100; α4, 105–128; α5, 132–141; α9, 249–265; α10, 268–281; η2, 289–296), two N-terminal β-strands (β1, 29–33; β2, 36–39), and a single C-terminal β-strand (β7, 284–286). Furthermore, the streptococcal PrsA protomers are linked by a domain swap; protomers A and D each donate a segment of α1 and the β1, β2, and β7 strands to the domain swap (13, 35). The interprotomer, three-stranded β-sheets formed by β1, β2, and β7 strands are presumed to interact with the cell membrane (13).

**Fig 2:**
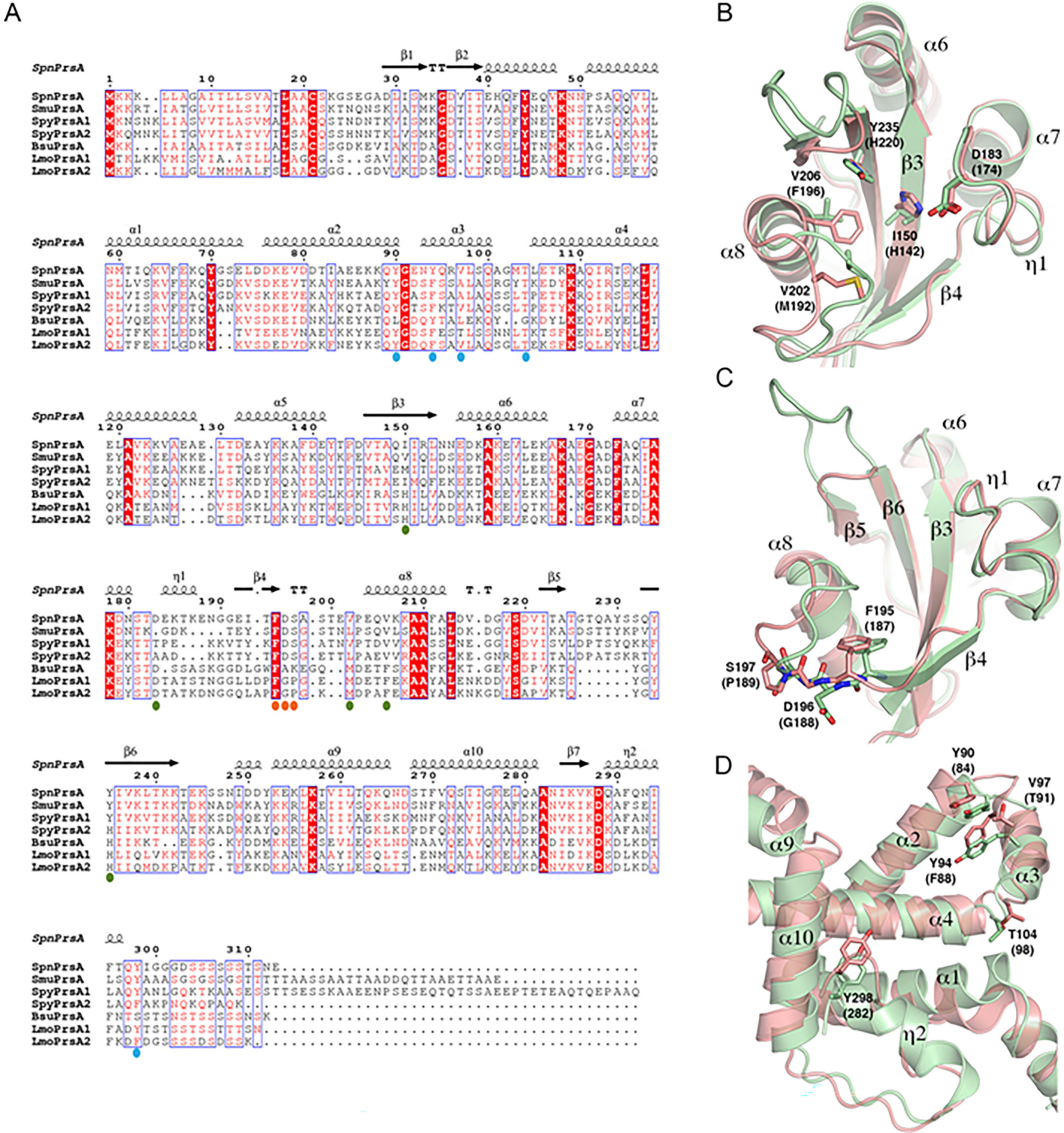
A comparison of PrsA structures from gram-positive bacteria. (**A**) PrsA sequences from *S. pneumoniae* (*Spn*PrsA; PDB 5TVL), *S. mutans* (*Smu*PrsA; PDB 7L75), *S. pyogenes* PrsA1 (*Spy*PrsA1; WP_011184661.1) and PrsA2 (*Spy*PrsA2; WP_010922719.1), *B. subtilis* (*Bsu*PrsA; PDB 4WO7), and *L. monocytogenes* PrsA1 (*Lmo*PrsA1; PDB 5HTF) and PrsA2 (*Lmo*PrsA2; WP_003724025.1) were used to generate a multiple sequence structural alignment in PROMALS3D (38), and the figure was rendered using ESPRIPT (39). The secondary structural elements of *Spn*PrsA (PDB 5TVL) are displayed above the sequences. Residues predicted to be involved in substrate binding and catalysis in the PPIase domain are marked by orange and olive spheres, respectively. Residues of the N and C domains predicted to be involved in foldase activity are marked by blue spheres. (**B and C**) Structural overlay of the parvulin domains of *Spn*PrsA (carbon backbone colored pale green) and *Lmo*PrsA1 (carbon backbone colored salmon). Key putative active site residues are shown as sticks and labeled according to *Spn*PrsA with the corresponding residues in *Lmo*PrsA1 in parentheses. Oxygen atoms are colored red, nitrogen are blue, and sulfur are yellow. (**D**) A structural overlay of the *Spn*PrsA and *Lmo*PrsA1 foldase domains with residues predicted to be involved in foldase activity shown as sticks. The structures are colored as in B and C.

When compared to homologs in *Bacillus subtilis* (*Bs*PrsA, PDB 4WO7) and *L. monocytogenes* (*Lmo*PrsA1*,* PDB 5HTF), both *Spn*PrsA and *Smu*PrsA are structurally similar to these known lipoprotein chaperones (Fig. 1C and 2A) (13, 35). Overlay of *Spn*PrsA with *Lmo*PrsA1 and *Bs*PrsA yields an r.m.s.d. of 2.99 Å across 249 residues and 2.20 Å across 250 residues, respectively (Fig. 1C). PrsA homologs are found in all streptococcal species and often contain a C-terminal intrinsically disordered region of unknown function (Fig. S3A). In addition, this PrsA C-terminal region is often enriched in serine residues at positions 303–308 (Fig. S3B). In PrsA homologs from some species, such as *S. pyogenes* PrsA1 and *Smu*PrsA, this C-terminal region is extended (Fig. 2A). As the structures of *Spn*PrsA and *Smu*PrsA align well with each other and PrsA structures from other gram-positive organisms, we anticipate that this domain architecture is conserved across most gram-positive organisms.

We compared the parvulin domains from *Spn*PrsA and *Lmo*PrsA1 (PDB 5HTF, r.m.s.d. of 1.71 Å) to analyze the *Spn*PrsA putative active site (Fig. 2B). Residues Ile150, Asp183, Val202, Val206, and Tyr235 of *Spn*PrsA correspond to catalytic residues His142, Asp174, Met192, Phe196, and His220 of *Lmo*PrsA1. These catalytic site residues are conserved in *Bs*PrsA, which, like *Lmo*PrsA homologs, displays PPIase activity (35, 40). Strikingly, only the catalytic site Asp is conserved in *Spn*PrsA. Moreover, both His residues from *Lmo*PrsA homologs are replaced by hydrophobic residues in *Spn*PrsA (I150 and Y235), and canonical hydrophobic characteristics are maintained at Val202 (Met192) and Val206 (Phe196), albeit with smaller, aliphatic residues. Thus, there are more hydrophobic components in the *Spn*PrsA putative active site compared to the *Lmo*PrsA homologs. Interestingly, despite an apparent increased hydrophobicity of the putative active site residues across streptococcal PrsA proteins compared to other gram positives, the catalytic site Asp residue is not conserved within the genus (Fig. 2A). There are also stark differences in residues at the predicted substrate binding site between *Spn*PrsA and *Lmo*PrsA homologs (Fig. 2C). Although Phe195 is present in all homologs examined, Asp196 and Ser197 are conserved in streptococcal species but diverge significantly from their corresponding residues in *Lmo*PrsA homologs and other gram-positive organisms.

We also compared the *Spn*PrsA foldase domain to that of *Lmo*PrsA1 (r.m.s.d. of 3.85 Å). This domain contains multiple residues important for the function of *Lmo*PrsA1 and *Lmo*PrsA2 in antibiotic, ethanol, and lysozyme resistance, in addition to the function of *Lmo*PrsA2 in virulence (13). Unlike the PPIase domain, *Spn*PrsA and *Lmo*PrsA homologs share more similarity across residues in the foldase (N) domain thought to be important for foldase activity, including Tyr90, Try94, Val97, Thr104, and Try298 (Fig. 2D). In all, streptococcal PrsA proteins share significant structural similarity with each other and PrsA from other gram-positive bacteria, but key amino acid differences across genera may be indicative of functional differences, including substrate specificity, between organisms.

### Structural comparison of streptococcal SlrA to other cyclophilins

**Fig 3:**
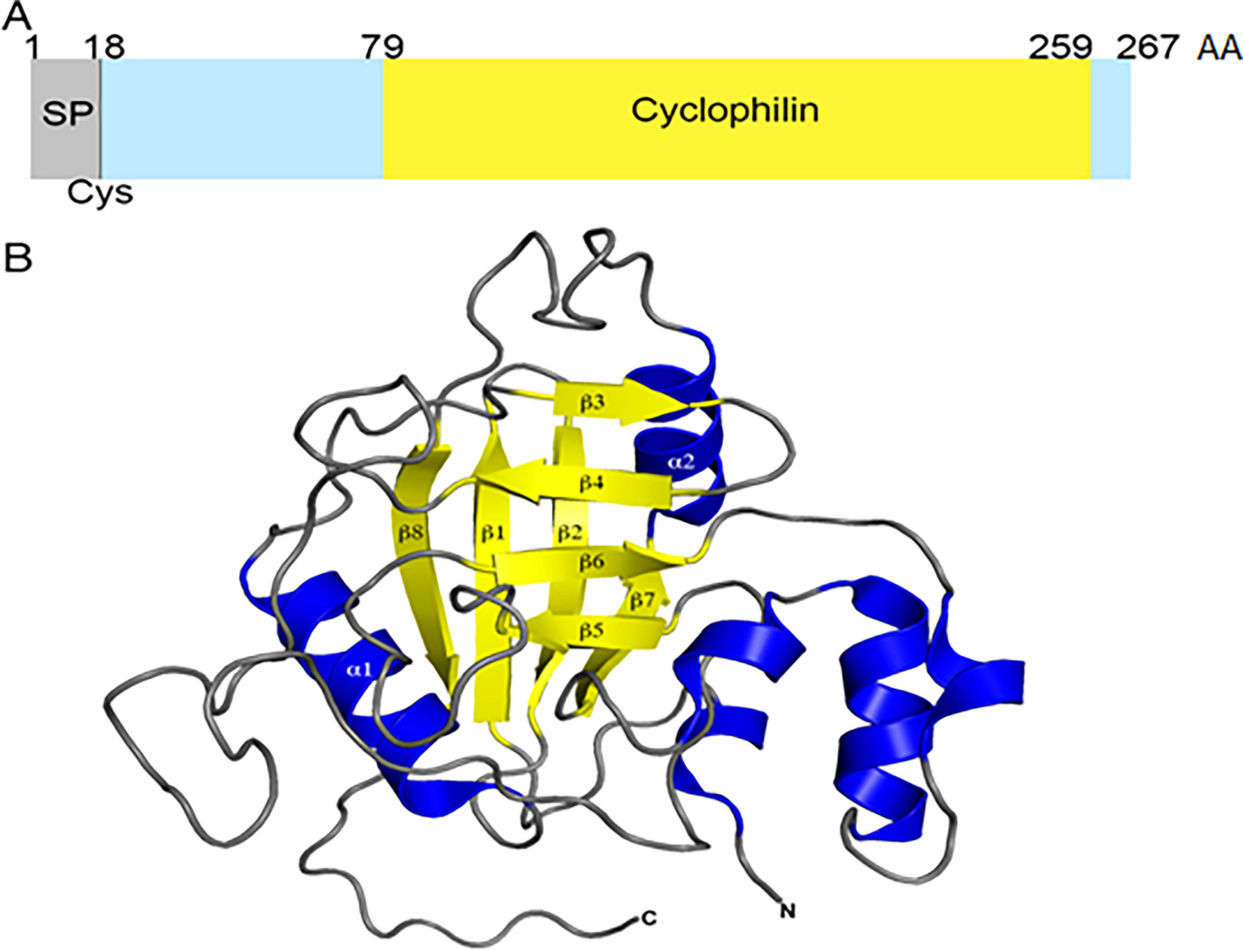
Overall structure of SlrA (PpiA) from gram-positive bacteria. (**A**) *S. pneumoniae* SlrA (*Spn*SlrA) domain organization was generated with InterProscan (36). The signal peptide (SP), the conserved lipid-modified cysteine (Cys, residue 18) (37), the N- (residues 19–79) and C-terminal (residues 260–267) domains, and the cyclophilin PPIase domain (residues 80–259) are indicated. (**B**) The overall structure of *Spn*SlrA (PDB 7L6Z, chain A) depicted as a cartoon with helices in blue, β-strands in yellow, and loop regions in gray. Canonical secondary structural elements are labeled according to equivalent regions in the homologous structure from human cyclophilin A.

To characterize the streptococcal cyclophilins, we first determined the domain organization of the SlrA-like proteins using InterProscan (36) (Fig. 3A). The server predicts the presence of a central cyclophilin domain flanked on its N- and C-termini with additional secondary structural elements. We then solved the crystal structures of SlrA homologs from *S. pneumoniae* (*Spn*SlrA) and *S. pyogenes* (*Spy*PpiA) to 1.88 Å and 1.28 Å resolution, respectively, using single-wavelength anomalous diffraction (SAD) data from selenomethionine derivatized crystals (Fig. 3B; Fig. S4A and B). All chains were refined independently, and the data quality and refinement statistics are listed in Table 2.

**TABLE 2:** Data quality and refinement statistics^*a*^

Statistic	Value for structure with PDB accession code:	
	7L6Y	7L6Z
Data collection		
Space group	*C2*	*P2_1_*
Unit cell parameters (Å; °)	*a* = 68.25, *b* = 50.53, *c* = 106.98; *α = 90.00, β = 92.32, γ = 90.00*	*a* = 88.43, *b* = 156.53, *c* = 90.54; α = 90.00, β = 117.18, γ = 90.00
Resolution range (Å)	30.00–1.28 (1.30–1.28)	30.00–1.88 (1.91–1.88)
No. of reflections	92,003 (4,451)	175,455 (8,741)
*R*_merge_ (%)	7.9 (79.9)	8.7 (75.8)
Completeness (%)	98.3 (96.5)	99.1 (99.1)
⟨*I*/*σ*(*I*)⟩	37.1 (3.0)	16.6 (2.3)
Multiplicity	7.9 (7.7)	4.4 (4.4)
Wilson *B* factor	14.4	27.3
Refinement		
Resolution range (Å)	27.12–1.28 (1.31–1.28)	29.97–1.88 (1.93–1.88)
Completeness (%)	98.1 (95.1)	98.9 (97.7)
No. of reflections	87,382 (4,603)	166,357 (12,125)
*R*_work_/*R*_free_, (%)	15.8/19.5 (23.1/26.8)	17.3/27.3 (21.2/30.2)
Protein chains/atoms	2/3,422	10/16,741
Ligand/Solvent atoms	0/504	107/1528
Mean temperature factor (Å^2^)	20.0	36.0
Coordinate deviations		
R.m.s.d. bonds (Å)	0.006	0.005
R.m.s.d. angles (°)	1.409	1.329
Ramachandran plot		
Favored (%)	97.0	96.0
Allowed (%)	3.0	4.0
Outside allowed (%)	0.0	0.0

^*a*^ Values in parentheses are for the outer shell.

The final model of *Spn*SlrA (PDB 7L6Z) contains 10 polypeptide chains, seven chloride anions, one 1,2-ethandiol molecule, eight 2-(N-morpholino)-ethanesulfonic acid (MES) molecules, and 1,532 water molecules (Fig. 3B; Fig. S5A and B). Models for two chains (A, D) contain all residues of the recombinant protein: an N-terminal methionine, residues 61–267 of *Spn*SlrA, and six residues of the fused C-terminal His-tag (Fig. S5A and B). For the remaining eight chains, the electron density maps had insufficient quality to model the C-terminal residue. A pairwise alignment of the 10 chains in this model showed that chains C and F had the lowest r.m.s.d. (0.20 Å) between Cα atoms, whereas the highest r.m.s.d. (1.28 Å) observed was between chains D and H or E and H.

The final model of *Spy*PpiA (PDB 7L6Y) contains two polypeptide chains and 521 water molecules. Although the expression construct encodes residues 21–268, three residues of the purification tag and 39 N-terminal residues were disordered in the crystal; thus, only residues 60–268 could be resolved (Fig. S4A). Superimposition of chains A from the *Spn*SlrA and *Spy*PpiA structures confirms that their structures are homologous and share good alignment with an r.m.s.d. of 2.08 Å (Fig. S4B). The *Spn*SlrA and *Spy*PpiA proteins have a canonical cyclophilin fold comprised of eight antiparallel β-strands (*Spn*SlrA: β1, 72–81; β2, 82–90; β3, 112–120; β4, 121–127; β5, 166–173; β6, 181–188; β7, 221–227; β8, 250–260) that form a β-barrel with two α-helices (*Spn*SlrA: α1,95–108; α2, 230–240) located at the top and the bottom of the barrel (Fig. 3B; Fig. S4A and B). Both structures also contain extra secondary structural elements adjacent to the β-barrel composed of residues 189–212 (η2, 193–195; η3, 203–211; η4, 215–217). Since there are no structures available for other streptococcal PpiA proteins, the structures presented here are the first for gram-positive cyclophilin-like PPIases.

**Fig 4:**
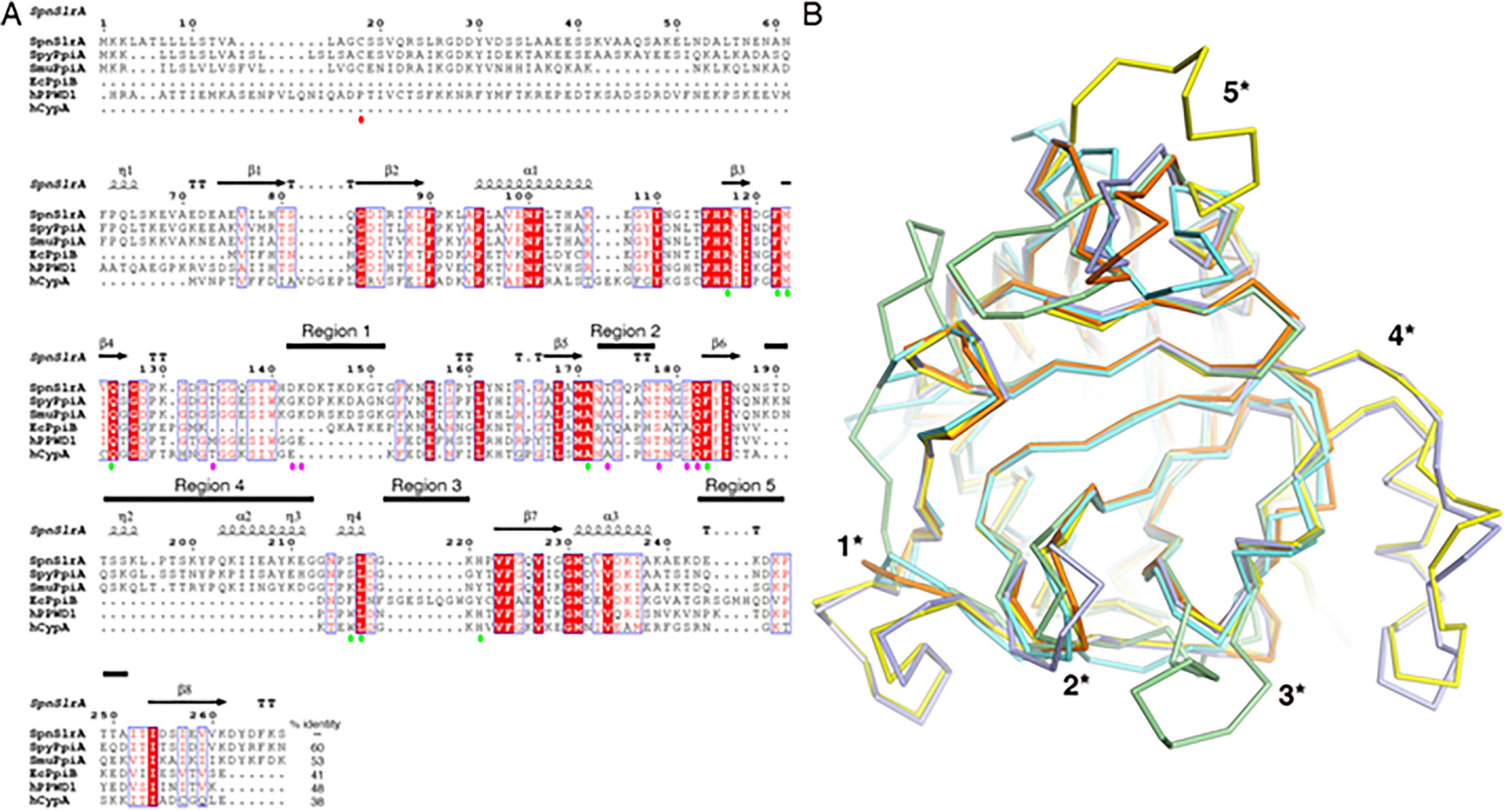
Structural comparison between gram-positive bacteria, gram-negative bacteria, and human cyclophilin proteins. (A) Structure-guided sequence alignment of *Spn*SlrA (AAK99483), *Spy*PpiA (AAZ50989), *Smu*PpiA (WP_002263610), *E. coli* PpiB (*Ec*PpiB; NP_415057), human PPWD1 (hPPWD1; WP_000477225), and hCypA (NP_001265855) generated with PROMALS3D (38) and rendered using ESPRIPT (39). The secondary structural elements and residue numbers corresponding to *Spn*SlrA are mapped above the alignment. The regions that differ across superimposed structures are marked by black bars above secondary structure elements. Active site and “gatekeeper” residues identified in hCypA (42) are marked by lime green and magenta spheres, respectively. The percent identity of primary amino acid sequence is provided as determined by Clustal Omega (43). (B) Ribbon representation of the structural alignment between *Spn*SlrA (PDB 7L6Z, violet), *Spy*PpiA (PDB 7L6Y, yellow), hPPWD1 (PDB 2A2N, orange), hCypA (PDB 1CWA, cyan), and *Ec*PpiB (PDB 1LOP, pale green). Regions with significant conformational differences are labeled as 1* (β4–β5 loop); 2* (β5–β6 loop); 3* and 4* (β6–β7 loop); and 5* (α2–β8 loop).

As expected, searches using the DALI Protein Structure Comparison Server (41) with the *Spn*SlrA and *Spy*PpiA structures show that they are similar to human cyclophilins and to *Escherichia coli* cyclophilins despite significant variation at the primary sequence level (Fig. 4A). Secreted SlrA homologs are conserved across streptococcal species, as *Spn*SlrA shares 60% and 53% sequence identity with *Spy*PpiA and *Smu*PpiA, respectively. Cytoplasmic SlrA homologs are found in gram-negative organisms, like PpiB in *E. coli* (PDB 1LOP) which shares 41% identity with *Spn*SlrA. Lastly, the human PPIases, peptidyl-prolyl isomerase containing WD40 repeat protein 1 (PPWD1, PDB 2A2N) and human cyclophilin A (hCypA, PDB 1CWA), share 48% and 38% identity with *Spn*SlrA, respectively. A multi-sequence structural alignment (Fig. 4A) and structural superimpositions (Fig. 4B) of our SlrA structures with known homologs emphasize the conservation of the fold across cyclophilin-type PPIases from distantly related organisms, but also reveal five structural regions of interest.

Differences between gram-positive SlrA homologs and other cyclophilins exist in regions 1–5 (Fig. 4A and B). Streptococcal SlrA proteins contain a conserved 9-residue insertion in the β4–β5 loop, region 1, and a 24-residue insertion in the β6–β7 loop, region 4. In streptococcal SlrA homologs, the region 4 insertion forms a structured appendage in the β6–β7 loop, composed of helices η2, η3, and η4. Interestingly, *E. coli* PpiB also has an eight-residue insertion in this loop, labeled as region 3; however, its orientation is different (Fig. 4A and B). Structural similarity search using VAST+ (44, 45) suggests that the 9- and 24-residue insertions introduced a novel protein fold in secreted streptococcal cyclophilins. As the insertions in β4–β5 and β5–β6 loops are conserved in multiple bacterial cyclophilin-like PPIases, they are likely to have some functional significance. To gain further insights into these novel structured appendages/insertions, we utilized AlphaFold3 (46) to model our solved *Spn*SlrA protein structure and a known tetrapeptide substrate (amino acids AAPF) of the human cyclophilin A (Fig. S6A and B). The predicted interaction revealed a model where the appendages interact with the AAPF substrate in a dynamic fashion, suggesting that these appendages may aid in substrate recognition or activity (Fig. S6A and B).

Compared to regions 1 and 4, regions 2 (β5–β6 loop) and 5 (α2–β8 loop) are likely conformational changes introduced by natural sequence variation instead of large insertions. The β5–β6 loop is similar across all species examined despite a single residue insertion in the *Spy*PpiA and *E. coli* PpiB sequences. The α2–β8 loop of *Spn*SlrA, *Spy*PpiA, and human cyclophilins are found in different conformations and are composed of fewer residues than *E. coli* PpiB. This variation might correspond to differences in protein peptide binding as observed in other cyclophilin-like PPIases (42).

### Comparison of the streptococcal SlrA and human cyclophilin PPIase domain active sites

The human cyclophilin, hCypA, has been characterized at the biochemical and structural levels (42). Residues found in the β4–β5 and the β5–β6 loops contribute to substrate binding as they form an expanded active site called the S2′ pocket that binds the residues downstream of the substrate proline, and residues thought to act as “gatekeepers” of substrate binding (Fig. 4A). Some of these residues vary across human proteins, whereas others are much more conserved (42). In addition, the hCypA active site includes an unvarying catalytic Arg, and several other residues (Phe, Met, Gln, Trp, Leu and His) that converge to form the S1′ pocket where the target proline binds (Fig. 4A). Many of these S1′ residues are identical across the human PPIase family (42). As *Spn*SlrA and *Spy*PpiA structures are significantly similar to hCypA (PDB 1CWA) with an r.m.s.d. of 1.54 Å across 147 Cα and 1.83 Å across 154 Cα, respectively, we closely examined the residues in and around their putative active sites (Fig. S6C).

**Fig 5:**
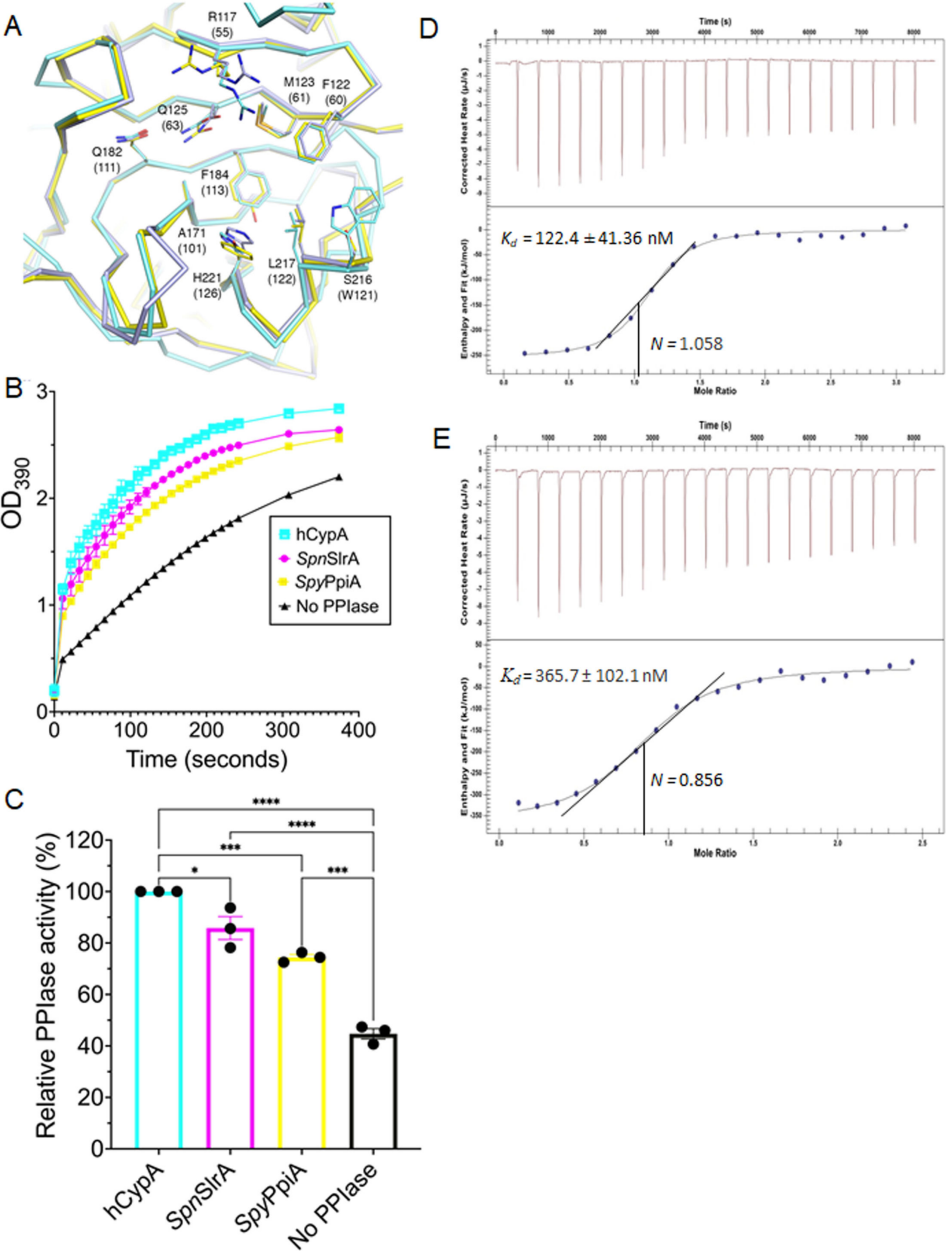
Comparison of PPIase characteristics between gram-positive and human cyclophilins. (**A**) Superposition of the carbon backbone of the putative active sites of *S. pneumoniae* SlrA (*Spn*SlrA, violet), *S. pyogenes* (*Spy*PpiA, yellow), and hCypA (cyan) (42). The putative active site residues are shown as sticks; oxygens, red; nitrogen, dark blue; sulfurs, orange. Residues are numbered according to *Spn*SlrA residues and in parentheses according to hCypA, using single letter code. (**B**) Protease-coupled PPIase activity using purified 6-His-tagged *Spn*SlrA and *Spy*PpiA in comparison to recombinant hCypA (*E. coli* derived). hCypA (⬜), *Spn*SlrA (●), and *Spy*PpiA (■) all demonstrate PPIase activity. Assays were performed with cyclophilins at constant concentrations of 10 µM with approximately 100 µg/mL Suc-Ala-Ala-Pro-Phe-pNA tetrapeptide substrate, compared to a negative control (No PPIase enzyme ▲). (**C**) The relative PPIase activity of *Spn*SlrA, *Spy*PpiA, and no-PPIase control are compared to hCypA (after approximately 400 seconds) and the activity of the PPIases are also compared to the no-PPIase control using one-way analysis of variance with Dunnett’s multiple comparisons test, *P < 0.01, ***P < 0.001, ****P < 0.0001, error bars represent the standard error of the mean of three independent experiments. (D and E) Binding interaction between *Spn*SlrA, *Spy*PpiA, and cyclosporine A (CsA) were determined using affinity isothermal titration calorimetry (ITC). SlrA (97.0 µM) in the syringe was titrated against CsA (5 µM) in the sample cell and PpiA (87.0 µM) in the syringe was titrated against CsA (6.5 µM) in the sample cell. For SlrA, the solid line corresponds to the fitted curve with a molar ratio of approximately 1:1 (*N* is 1.058). The *K*_*d*_ is 122.4 ± 41.36 nM, Δ*H* is −254.8 ± 10.97 kJ/mol, *K*_*a*_ is 8.167 × 10^6^ M^−1^, Δ*G* is −39.45 kJ/mol, and Δ*S* is −722.2 J/mol·K. For PpiA, the solid line corresponds to the fitted curve with a molar ratio of approximately 1:1 (*N* is 0.856). The *K*_*d*_ is 365.7 ± 102.1 nM, Δ*H* is −356.0 ± 18.04 kJ/mol, *K*_*a*_ is 2.735 × 10^6^ M^−1^, Δ*G* is −36.74 kJ/mol, and Δ*S* is −1071 J/mol·K. Both ITC thermograms are representative graphs of three independent experiments for each chaperone.

Residues Thr134, Lys143, Thr178, Ser181, and Gln182 in *Spn*SlrA correspond to human “gatekeeper” residues and are similar across bacterial and human cyclophilin-like PPIases (Fig. 4A). However, *Spn*SlrA Asp142 and its corresponding residues are more variable across organisms (Fig. 4A). In hCpyA, the side chains of these “gatekeeper” residues control access to the S2′ pocket. We suspect that these residues serve similar functions in streptococcal cyclophilin-like PPIases. A comparison of the *Spn*SlrA and *Spy*PpiA putative active sites with that of hCypA showed that consistent with previous reports, a catalytic Arg117 is conserved in these proteins, although their orientations differ slightly (Fig. 5A). Furthermore, residues corresponding to *Spn*SlrA Phe122, Met123, Gln125, Ala171, and Leu217 are all identical in *Spy*PpiA and hCypA (Fig. 5A). Interestingly, in *Spn*SlrA, Phe184 at the base of the S1′ pocket and His221 at the side of the S1′ pocket are identical to hCypA residues, but both are replaced by Tyr in *Spy*PpiA (Fig. 5A).

Previous characterization of human cyclophilin PPIase activity demonstrated that only proteins with Trp, Tyr, or His at the position corresponding to *Spn*SlrA residue 216, an important substrate binding determinant, were functional (42). Furthermore, when Hermans et al. demonstrated that *Spn*SlrA functions as a PPIase (15), in the absence of its structure, they suggested that Tyr was important for catalysis. Our structure-based sequence alignments place a serine at this important position in homologs *Spn*SlrA and *Spy*PpiA, and a lysine is present at this position in the *S. mutans* homolog PpiA (Fig. 4A). Indeed, in structural superimpositions, both *Spn*SlrA and *Spy*PpiA substitute Trp with Ser216 (Fig. 5A). Although serine and lysine are not known to permit human cyclophilin PPIase activity, our structural alignments suggest that either of these might facilitate substrate binding and PPIase activity in streptococcal species.

### *S. pneumoniae* SlrA and *S. pyogenes* PpiA possess functional cyclophilin activity

As described above, *Spn*SlrA putative active site residues Phe184 and His221 are identical to hCypA residues but are substituted by Tyr in *Spy*PpiA (Fig. 5A). Thus, we sought to determine whether these substitutions still allow for *Spy*PpiA PPiase activity. *Spn*SlrA, *Spy*PpiA, and hCypA PPIase activity were measured using a modified protease-coupled chymotrypsin assay (47). *Spn*SlrA, *Spy*PpiA, and hCypA all catalyzed the *cis*-to-*trans* isomerization of the *N*-succinyl-Ala-Ala-Pro-Phe-*p*-nitroanilide substrate (Fig. 5B) (15, 42). However, we observed ~20% decrease in PPIase activity of *Spn*SlrA and *Spy*PpiA compared to hCpyA (Fig. 5C). These data indicate that Tyr substitutions at key putative active site positions allow for PPIase activity in, at least, one streptococcal cyclophilin. We consider that the observed differences in PPIase activity between streptococcal and human cyclophilins in our assay may result from differences in key residues in and around their putative active sites, including Ser216.

As cyclophilins were first characterized for their ability to bind the immunosuppressive drug cyclosporine A (CsA) (48), we asked whether *Spn*SlrA and *Spy*PpiA interacts with CsA. Using affinity isothermal titration calorimetry (ITC), we determined that *Spn*SlrA and *Spy*PpiA bind to CsA with a dissociation constants (*K*_*d*_) of 122.4 ± 41.4 nM and 365.7 ± 102.1 nM, respectively (Fig. 5D and E). The observed dissociation constants are within the range of affinities of human cyclophilins for CsA (42). Our results indicate that *Spn*SlrA and *Spy*PpiA interact with CsA with a stoichiometry of 1:1 (Fig. 5D and E). Although the inhibitory constant of *Spn*SlrA activity to CsA was previously determined (15), this is the first time we observe the thermodynamic interactions between *Spn*SlrA and CsA and the first characterization of the interaction of *Spy*PpiA with CsA.

### *S. pneumoniae* extracellular ATP-independent chaperones are critical for pneumolysin maturation in secreted fractions

**Fig 6:**
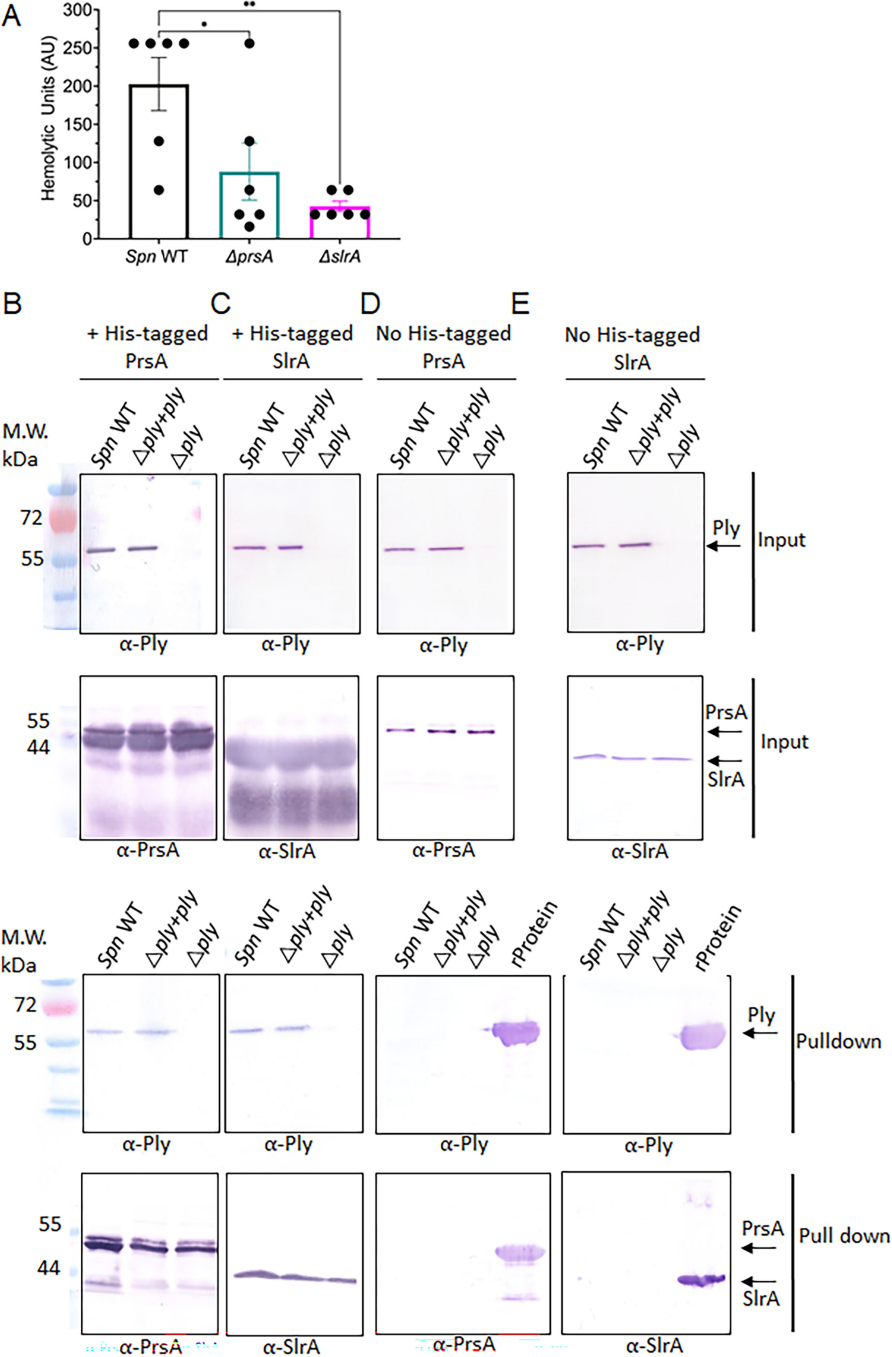
*Spn*PrsA and *Spn*SlrA interact with pneumolysin in cell wall fractions. (**A**) Bacterial cell wall fractions from *S. pneumoniae* wild-type (*Spn* WT), Δ*prsA,* and Δ*slrA* strains were assessed for the ability to lyse sheep’s red blood cells (RBCs). The reciprocal of the dilution that resulted in 100% RBC lysis (hemolytic units) was determined for three independent mutants conducted in duplicate. Error bars represent the standard error of the mean for the six assays. Asterisks (*) indicate statistical significance of *P* < 0.01 by one-way analysis of variance with Dunnett’s multiple comparisons test of mutant strains as compared to wild type. (**B–E**) Immunoblots of *S. pneumoniae* PrsA, SlrA, and Ply proteins from bacterial cell wall fraction protein pull-down assays. Hexa-his-tagged *Spn*PrsA or *Spn*SlrA (B and C, respectively) were added to cobalt beads where control experiments had no added hexa-his-tagged chaperones (D and E) and incubated with protein fractions from the bacterial cell wall. Input, proteins pulled down by the hexa-his-tagged *Spn*PrsA or *Spn*SlrA, and the elute of material recovered from experiments without hexa-his-tagged chaperones were subjected to SDS-PAGE followed by Western blotting analysis using antibodies (α) directed toward PrsA, SlrA, or Ply. For experiments where no hexa-his-tagged chaperones were added (D and E), a recombinant protein (rProtein) control was included on the SDS-PAGE followed by Western blotting for the pull-down.

Our characterization of the ATP-independent chaperones PrsA and SlrA indicates that these protein structures are conserved across streptococcal species and some of their functions may also be conserved across homologs from distantly related organisms. However, there remain gaps in our knowledge of streptococcal PrsA and SlrA substrates. PPIase homologs from *S. pneumoniae* and other organisms, including PrsA2 from *L. monocytogenes*, are known meditators of secreted virulence factor maturation (12, 24, 32, 49). Recently, we determined that Ply levels were altered in cell wall and released fractions of *S. pneumoniae prsA* and *slrA* deletion mutants (12); therefore, we tested whether streptococcal PrsA and SlrA are critical for the maturation of Ply. We assessed Ply activity by assaying the hemolytic units of cell wall and supernatant fractions from the *S. pneumoniae* strain TIGR4 ∆*prsA* and ∆*slrA* mutants as compared to the wild-type strain. We observed a significant reduction in hemolytic activity from cell wall fractions when *prsA* and *slrA* are deleted as compared to the wild-type *S. pneumoniae* strain TIGR4 (Fig. 6A). Consistent with these results and our previous proteomic data (12), there is less Ply protein in cell wall fractions of ∆*prsA* and ∆*slrA* mutants compared to the wild-type strain (Fig. S7). Additionally, the hemolytic activity and protein levels in supernatant fractions of ∆*prsA* and ∆*slrA* mutants are significantly reduced compared to wild-type cells (Fig. S8A and B), suggesting that PrsA and SlrA are critical for the maturation of the Ply toxin.

Since protein maturation can rely on chaperone association, we first determined whether a physical interaction exists between PrsA and/or SlrA and Ply using a chaperone pull-down assay. We immobilized cobalt beads with the recombinant hexa-his-tagged chaperones and added *S. pneumoniae* cell wall protein fractions. Strikingly, both PrsA and SlrA pulled down the Ply toxin in cell wall fractions suggesting a possible interaction between the chaperones and Ply (Fig. 6B and C). As a control, Ply was not detected by anti-Ply antibodies in the absence of hexa-his-tagged PrsA or SlrA chaperones (Fig. 6D and E). Therefore, PrsA and SlrA each interact with Ply in cell wall fractions and are required for full secreted Ply hemolytic activity in *S. pneumoniae*.

### *S. pneumoniae* PrsA and SlrA chaperones directly associate with the pneumolysin toxin

**Fig 7:**
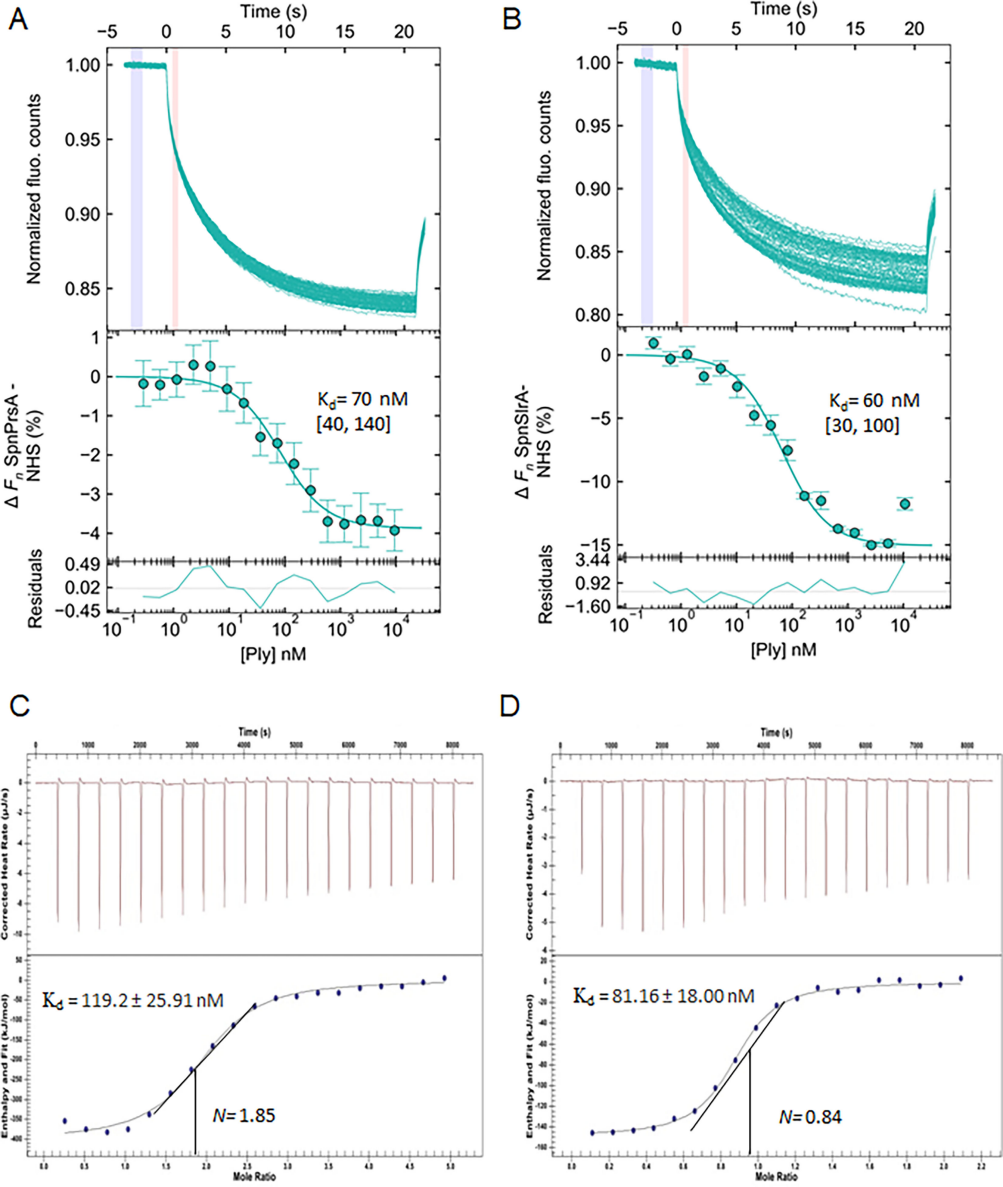
*Spn*PrsA and *Spn*SlrA demonstrate a direct interaction with pneumolysin. (**A and B**) The binding interaction between PrsA and SlrA with Ply was measured using MST. The concentration of the labeled chaperone was kept constant (20 nM), while the concentration of the serial dilution series of the non-labeled Ply ligand varied (from 180 nM to 9.4 µM for reactions with PrsA and 140 nM to 8 µM for reactions with SlrA). MST measurements were analyzed using PALMIST (50) and figures were plotted using GUSSI v.1.2.0 (51). The top panel shows the thermophoretic time-traces of three or more experiments, and the blue and pink areas represent time spans used to obtain the fluorescence cold (*F*_*c*_) and hot (*F*_*h*_) regions, respectively. The middle panel shows the binding curve with the line of best fit using the 1:1 binding model (95% CI), and the error bars represent the standard deviation. The residuals between the data and fit are shown in the bottom panel. The PrsA dissociation constant (*K*_*d*_) is 70 nM [40, 140] and the SlrA *K*_*d*_ is 60 nM [30, 100] to Ply, respectively. (**C and D**) The binding interaction between PrsA and SlrA with Ply was measured using affinity ITC. PrsA (50.8 µM) in the syringe was titrated against Ply (1.4 µM) in the sample cell and SlrA (66 µM) in the syringe was titrated against Ply (5 µM) in the sample cell. For PrsA, the solid line corresponds to the fitted curve with a molar ratio of approximately 2:1 (*N* is 1.85). The *K*_*d*_ is 119.2 ± 25.91 nM, Δ*H* is −419 ± 15.65 kJ/mol, *K*_*a*_ is 8.38 × 10^6^ M^−1^, Δ*G* is −39.52 kJ/mol, and Δ*S* is −1274 J/mol·K. For SlrA, the solid line corresponds to the fitted curve with a molar ratio of approximately 1:1 (*N* is 0.84). The *K*_*d*_ is 81.16 ± 18.00 nM, Δ*H* is −149.0 ± 4.055 kJ/mol, *K*_*a*_ is 1.232 × 10^7^ M^−1^, Δ*G* is −40.47 kJ/mol, and Δ*S* is −364.0 J/mol·K. Both ITC thermograms are representative graphs of three independent experiments.

Since our *S. pneumoniae* PrsA and SlrA pull-down assays indicate that both chaperones interact with Ply, we tested whether recombinant *Spn*PrsA and/or *Spn*SlrA directly bind to Ply using microscale thermophoresis (MST) at pH 6.5 where the Ply homolog LLO exists in a partially folded state (31) with the rationale that chaperones interact with loosely or unfolded substrates. For MST experiments, we labeled purified recombinant PrsA and SlrA with the amine-reactive fluorophore N-hydroxysuccinimide (NHS) and utilized these NHS-labeled proteins to detect an interaction with Ply. Our results indicate that PrsA and SlrA bind to Ply with physiologically relevant affinities, *K*_*d*_ of 70 [40, 140] nM and 60 [30, 100] nM, respectively (Fig. 7A and B).

To confirm the binding of PrsA and SlrA to Ply without the use of fluorophores, which can potentially interfere with the interaction, we used affinity ITC with recombinant purified unlabeled proteins. As Ply is slowly titrated into a cell containing *Spn*PrsA or *Spn*SlrA, the change in raw heat of the system gives an interaction affinity *K*_*d*_ of 119.2 ± 25.9 nM and 81.16 ± 18.00 nM for PrsA and SlrA, respectively, with Ply (Fig. 7C and D). Both ITC and MST results demonstrate the interaction of Ply with both *Spn*PrsA and *Spn*SlrA with a comparable *K*_*d*_. Our ITC results indicate that the molar ratio for PrsA to Ply is 2:1, suggesting that one PrsA dimer interacts with one Ply monomer. The molar ratio for SlrA to Ply is approximately 1:1, indicating that one SlrA monomer interacts with one Ply monomer.

To delineate domains of *Spn*PrsA that facilitate its interaction with Ply, we determined the affinity of a recombinant *Spn*PrsA N+C mutant (PrsA without the PPIase domain containing only the foldase domain) for Ply using ITC. Compared to the wild-type *Spn*PrsA ITC results, the *Spn*PrsA N+C exhibited poor affinity (*K*_*d*_ of 791.9 ± 103.0 nM) to Ply, an approximately 5.64-fold reduction, suggesting that the PPIase domain of PrsA is critical for binding to Ply (Fig. S9). To rule out whether the poor affinity of *Spn*PrsA N+C is due to protein structure instability, we performed differential scanning fluorimetry (DSF) where recombinant *Spn*PrsA and *Spn*PrsA N+C proteins were heated in the presence of a hydrophobic dye over a 65°C range. DSF of *Spn*PrsA and *Spn*PrsA N+C indicates both proteins show similar melting curves of 54.94 ± 0.18°C and 54.87 ± 0.08°C, respectively, suggesting similar thermal stability (Fig. S10A and B). We also analyzed the secondary structure of *Spn*PrsA and *Spn*PrsA N+C using circular dichroism (CD) spectroscopy which provides evidence that *Spn*PrsA and *Spn*PrsA N+C possess stable secondary structures (Fig. S10C).

To test the substrate specificity of *Spn*PrsA and *Spn*SlrA and provide further evidence that Ply is a cognate substrate of these chaperones, we determined the binding affinity by MST of *Spn*PrsA and *Spn*SlrA to non-specific substrates, bovine serum albumin (BSA) and lysozyme. *Spn*PrsA interacted with poor affinity to both BSA and lysozyme with a *K*_*d*_ of 62.0 ± 58 µM and 170 ± 10 µM, respectively (Fig. S11A and B). This indicates that *Spn*PrsA bound to BSA and lysozyme with approximately 8.85 × 10^2^-fold and 2.43 × 10^3^-fold reduced affinity, respectively, when compared to Ply. Similarly, *Spn*SlrA also bound with poor affinity to BSA and lysozyme with *K*_*d*_ of 6.8 ± 2.1 µM and 17.0 ± 1.5 µM, respectively (Fig. S11C and D). Therefore, *Spn*SlrA bound BSA and lysozyme with approximately 1.12 × 10^2^-fold and 2.82 × 10^2^-fold reduced affinity, respectively, when compared to Ply. Overall, although PrsA and SlrA interact with BSA and lysozyme, the observed binding affinities are several orders of magnitude less than the affinities observed for Ply. Taken together, these data suggest that both PrsA and SlrA interact with Ply with physiological affinities.

### *S. pneumoniae* extracellular ATP-independent chaperones accelerate the folding of the pneumolysin

**Fig 8:**
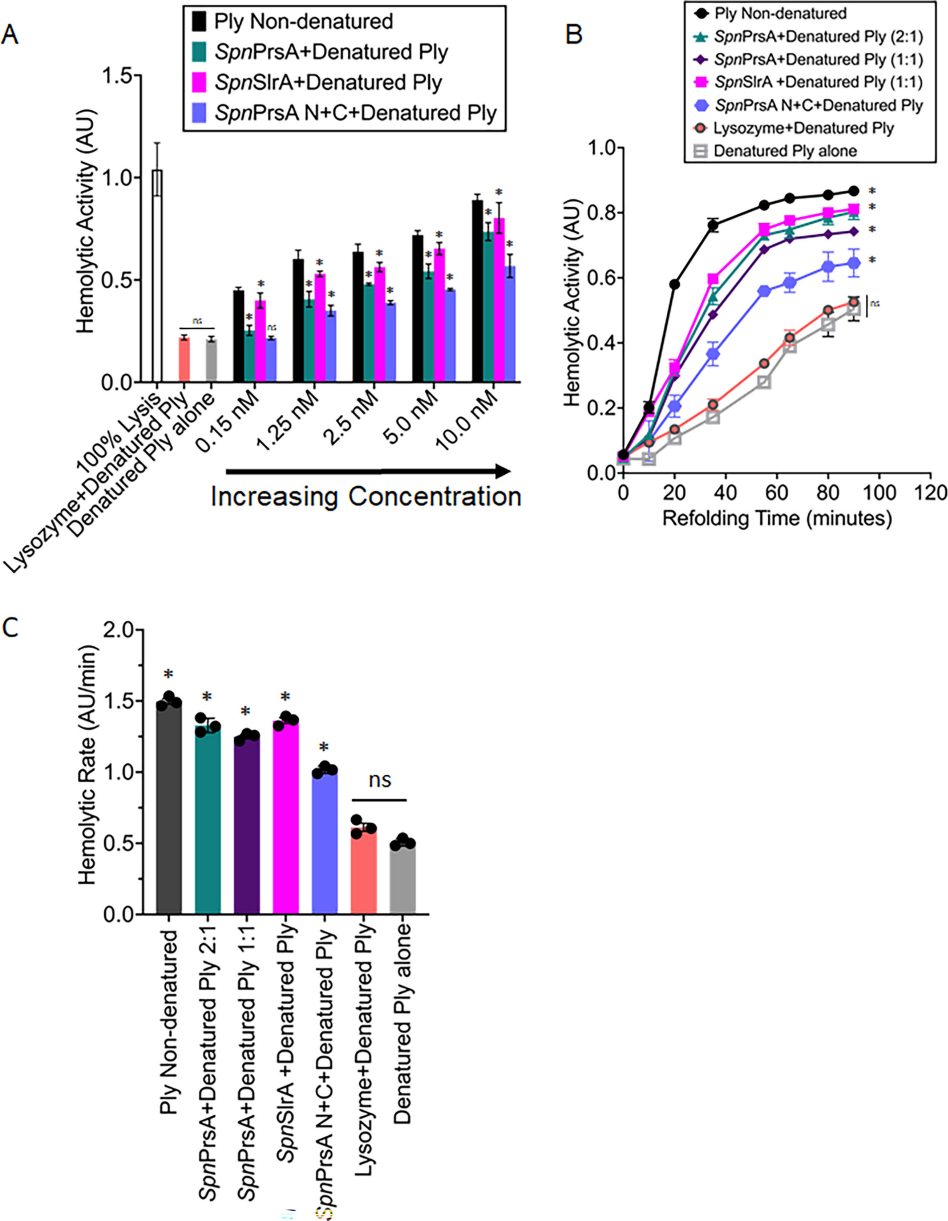
*Spn*PrsA and *Spn*SlrA accelerate the folding of the pneumolysin toxin. (**A**) Chaperone-assisted folding assay of denatured Ply alone or in the presence of PrsA, PrsA N+C, SlrA ,or lysozyme. The denatured Ply (10 nM) concentration remained constant while the non-denatured Ply alone control ranged from 0.15 nM to 10 nM as indicated. Hemolytic activity in the presence of PrsA, PrsA N+C, SlrA, or lysozyme (10 nM) was compared to the denatured Ply alone using one-way analysis of variance (ANOVA) with Dunnett’s multiple comparisons test where *P < 0.01. (**B**) Time-course hemolytic activity of denatured Ply in the presence of PrsA, PrsA N+C, SlrA ,or lysozyme using a stoichiometry of 1:1 and 2:1 chaperone to denatured Ply. Non-denatured Ply (10 nM), denatured Ply (10 nM), and denatured Ply (10 nM) with lysozyme (10 nM) are controls. Hemolytic rates of the chaperones were compared to the denatured Ply-alone sample using one-way ANOVA with Dunnett’s multiple comparisons test at 65 and 90 min, *P < 0.01. (**C**) Hemolytic rates of PrsA, PrsA N+C, SlrA, and lysozyme. The rates were determined by dividing the hemolytic activity by time at the 1 hour mark in B. Sample hemolytic rates were compared to the denatured Ply-alone sample using one-way ANOVA with Dunnett’s multiple comparisons test, *P < 0.01. Error bars represent the standard error of the mean for all experiments. Data represent three independent experiments and non-significant (ns) differences are indicated.

The observed interaction and reduction in hemolytic activity in *S. pneumoniae* Δ*prsA* and Δ*slrA* strains (Fig. 6A through E and 7A through D) suggest that PrsA and/or SlrA may accelerate the folding of Ply to promote maturation and downstream toxin function. To test whether PrsA or SlrA accelerate the folding of the Ply toxin, we developed a chaperone-assisted folding assay that measures the functional activity of Ply in the form of hemolytic activity, given that folded and active Ply has the ability to lyse red blood cells (RBCs) (52). For this assay, purified recombinant Ply is denatured in buffer containing 8 M urea, then diluted 20-fold and incubated in the presence and absence of the chaperones for 5 min. Next, sheep’s red blood cells are added, and Ply-dependent hemolytic activity is determined by measuring the amount of released hemoglobin. We observe that the addition of both *Spn*PrsA and *Spn*SlrA to denatured Ply results in a dose-dependent increase in Ply-dependent hemolytic activity compared to the denatured Ply alone control, suggesting that both chaperones fold Ply (Fig. 8A). In contrast, the addition of lysozyme did not increase the hemolytic activity of denatured Ply (Fig. 8A). *Spn*PrsA N+C also showed increased hemolytic activity as compared to the denatured Ply alone (Fig. 8A). However, the hemolytic activity of denatured Ply in the presence of *Spn*PrsA N+C is significantly reduced compared to the wild-type *Spn*PrsA protein, implying that the PPIase domain is important for Ply folding. Surprisingly, at high chaperone concentrations, we observed a reduction in the hemolytic activity of Ply, which suggests that both chaperones may possess an inhibitory or holdase activity (Fig. S12). As a control, lysozyme showed no effect at the same concentrations (Fig. S12).

*Spn*PrsA and *Spn*SlrA folding rates of Ply using observed ITC-measured stoichiometries of chaperone to Ply (Fig. 7C and D) indicate that both chaperones accelerate the folding of denatured Ply similarly from the 0 to 30 min time points (Fig. 8B and C). In contrast, the denatured Ply-alone control showed background hemolytic activity, suggesting that Ply can spontaneously fold at pH of 6.5 (Fig. 8B). As a control, the addition of lysozyme did not increase the hemolytic rate of denatured Ply (Figure 8; Fig. S12). In fact, lysozyme with denatured Ply was similar to the denatured Ply alone control. Additionally, we observe a reduction in the hemolytic rate of denatured Ply in the presence of PrsA N+C, suggesting that the PrsA PPIase domain is important for Ply activity (Fig. 8B and C).

Finally, although PrsA and SlrA interact with poor affinity to BSA and lysozyme, we considered the possibility that PrsA and SlrA probably only accelerate the folding of specific substrates such as the Ply. Therefore, we tested whether PrsA and/or SlrA showed a significant decrease in the folding rate of denatured Ply in the presence of BSA or lysozyme. We determined that BSA or lysozyme did not inhibit the ability of PrsA or SlrA to activate denatured Ply (Fig. S13), providing further evidence that PrsA and SlrA specifically recognize Ply and accelerate Ply folding. Together, these results suggest that both *Spn*PrsA and *Spn*SlrA bind and accelerate the folding of the Ply toxin.

## DISCUSSION

We determined the structures of ATP-independent PPIase chaperones from gram-positive bacteria in the genus *Streptococcus*. The parvulin-type PPIases, *Spn*PrsA and *Smu*PrsA, retain both PPIase and foldase domain architecture that has been observed in gram-positive PrsA homologs from *L. monocytogenes* and *B. subtilis* (13, 35). Previous work of *L. monocytogenes* PrsA homologs has shown that dimerization is critical for PrsA function (13). This is consistent with our dimeric *Smu*PrsA structure (Fig. S2A), as is the dimer of dimers observed in the *Spn*PrsA structure (Fig. S1A). In addition, although the *Spn*PrsA dimer of dimers may be a crystallization artifact, we do wonder whether this is evidence that PrsA may function in its own folding process during secretion. Indeed, the interface between these dimers is extensive (1,971 Å) (Fig. S1), suggesting that this form may reflect its solution state. The two cyclophilin-like streptococcal PPIases, *Spn*SlrA and *Spy*PpiA, are the first of such structures in gram-positive bacteria (Fig. 3B; Fig. S4A and B). These structures reveal the presence of unique structural appendages that may have roles in the recognition, binding, and folding of protein substrates (Fig. 4B; Fig. S6A and B).

Importantly, we have demonstrated that *Spn*PrsA and *Spn*SlrA contribute to the folding of the pneumococcal major virulence factor, Ply. Additionally, we showed that PrsA and SlrA possess poor binding affinity for non-specific substrates such as BSA and lysozyme (Fig. S11), probably due to the recognition of hydrophobic patches or certain physical and chemical properties (53, 54); however, PrsA and SlrA accelerate the folding of Ply even in the presence of BSA and lysozyme (Fig. S13). Although *Spn*PrsA lacks PPIase activity (15, 22), the parvulin PPIase domain of *Spn*PrsA is critical for binding and folding denatured Ply, suggesting that the *Spn*PrsA foldase and PPIase domains are important for Ply function (Fig. S9; Fig. 8). This lack of *Spn*PrA PPIase activity is likely the result of catalytic site residue differences Ile150, Val202, Val206, and Tyr235 that typically correspond to His, Met, Phe, and His, respectively, in gram-positive PrsA homologs that demonstrate PPIase activity. However, despite this lack of PPIase activity, pneumococcal *prsA* deletion mutants result in significant defects in virulence (12, 22, 23), suggesting that the PrsA protein plays a critical role in virulence.

Since PPIase activity has not been demonstrated for streptococcal PrsA homologs, the presence of secreted cyclophilin-like homologs that retain activity (Fig. 5B) suggests that PPIase activity is required for secreted protein folding and function (12). In addition, the putative active site residues of the streptococcal cyclophilin-like PPIases suggest conservation from bacteria to humans (Fig. 5A); therefore, the mode of enzymatic activity and substrate recognition may also be similar. However, the substitution of tryptophan with serine (S216) in *Spn*SlrA and *Spy*PpiA might suggest a difference in the types of substrates that are recognized by streptococcal cyclophilins (Fig. 5A). Our results show for the first time that *Spy*PpiA functions as a peptidyl-prolyl isomerase (Fig. 5B and C). However, tetrapeptide substrate preference is yet to be elucidated. Davis et al. have shown that the two primary active site pockets of the human cyclophilins in addition to an expanded pocket of “gatekeeper” residues are important for substrate specificity and access to the inner pockets (42). Although the primary pockets are conserved, the expanded pockets of “gatekeeper” residues in the streptococcal PPIases are variable and may be important for discrimination between substrates.

Surprisingly, a comparison of the *Spn*SlrA and *Spy*PpiA crystal structures with the human and *E. coli* cyclophilins revealed the presence of two structural appendages that are caused by 9- and 24-amino acid insertions (Fig. 4A and B). In addition, the two structural appendages contain lysine, serine, and aspartate residues and are conserved in most streptococcal species. The functional roles of these appendages are yet to be determined; however, because the amino acid residues of the structural appendages extend into the putative “gatekeeper” pocket, this region may be important for substrate selectivity or recognition.

For pathogenic streptococcal species to maintain virulence, they must secrete factors that allow them to evade host immune responses and promote bacterial growth and spread. During *S. pneumoniae* host infection, virulence factors are secreted from the cytosol into the space between the bacterial membrane and cell wall. Like other secreted proteins necessary for virulence, Ply is likely translocated across the bacterial membrane in an unfolded state. Since Ply lacks a signal sequence, others have determined that Ply is exported through a non-canonical mechanism (30) and/or through an accessory Sec system (55). Once exported, Ply was shown to associate with the cell wall of 18 different *S. pneumoniae* serotypes (29). Our results demonstrate that *S. pneumoniae* requires *prsA* and *slrA* for Ply maturation in cell wall and secreted fractions (Fig. 6A; Fig. S7, S8A and B). In addition, our results reveal that Ply is promiscuous based on its interaction with both *Spn*PrsA and *Spn*SlrA, suggesting Ply may require both PrsA foldase and SlrA PPIase activity (Fig. 6B through E and 7A through D). The observed interaction between Ply and both secreted ATP-independent chaperones further suggests Ply may be non-classically secreted. During *S. pneumoniae* infection, Ply assembles into rings of 30–50 subunits to form pores within host membranes (56). The rapid oligomerization of Ply may require multiple chaperones to quickly fold the toxin before it can form these pores. Our data show a robust decrease in the hemolytic activity of denatured Ply at high chaperone concentrations, suggesting that both chaperones may sequester folded Ply on the bacterial membrane interface as we observed for the listeriolysin O toxin (31).

Some gram-positive pathogens encode more than one PrsA homolog, for example, the PrsA1 and PrsA2 within *S. pyogenes* and *L. monocytogenes* (57). Deletion of *L. monocytogenes prsA2* results in several virulence defects while *L. monocytogenes prsA1* contributes to bacterial translocation across the intestine (32, 58). The role of *Lmo*PrsA1 during infection appears to be minor, whereas *Lmo*PrsA2 is required for mouse models of intragastric and septicemic infection, in addition to host cell-to-cell spread (32, 58). In *S. pyogenes*, the two PrsA homologs individually contribute to secreted protein homeostasis and share overlapping function in host adherence, biofilm formation, and virulence in a mouse infection model (14). But unlike *Lmo*PrsA1 and *Lmo*PrsA2, the two *S. pyogenes* PrsA homologs lack key PPIase catalytic active site residues and are not predicted to have PPIase activity (Fig. 2A); however, both extracellular chaperones are critical for bacterial virulence, suggesting that these chaperones play important roles in secreted virulence protein maturation and function.

The identification of specific PPIase chaperone substrates and the characterization of chaperone domains necessary for activity are critical for vaccine and therapeutic development due to the many potential interactions of PPIases with virulence factors (59–61). The X-ray crystal structures of several streptococcal PPIases in this study and the discovery of Ply as a substrate of both *Spn*PrsA and *Spn*SlrA is a step forward in understanding how secreted gram-positive PPIases help orchestrate protein folding and function post-membrane translocation during host infection. This knowledge may lead to the development of tools that block the activity of streptococcal PPIases or inhibit their interactions with virulence factors which may ultimately prevent the spread of bacterial pathogens. In conclusion, the data available thus far for streptococcal PPIases indicate their importance in virulence and, therefore, the potential of PPIase inhibition as an additional approach to antibiotics for treatment of streptococcal infections.

## MATERIALS AND METHODS

### Bacterial strains, plasmids, and media

Bacterial strains and plasmids used in this study are listed in Table S1. *S. pneumoniae* TIGR4 and mutants were grown in Todd-Hewitt broth supplemented with 0.5% yeast extract at 37°C with 5% CO_2_. *E. coli* strains were grown in Luria broth (LB) broth at 37°C or otherwise mentioned.

### Generation of *S. pneumoniae* mutants

*S. pneumoniae* Δ*prsA* (LAC-4417, LAC-4418, LAC-4419) and Δ*slrA* (LAC-4420, LAC-4421, LAC-4422) mutants used in this study were previously published (Table S2) (12). Briefly, tetracycline and spectinomycin resistance cassettes were ligated between ~1 kb upstream and downstream flanking sequences of *prsA* and *slrA*, respectively. These generated linear DNA fragments were transformed into the *S. pneumoniae* TIGR4 strain as previously described (12). Positive transformants were verified by PCR amplification and sequencing. *S. pneumoniae* TIGR4 Δ*ply* strains (Δ*ply::spc* and Δ*ply::spc + ply*) were a kind gift from Andrew Camilli at Tufts University and were also published previously (Table S2) (30).

### Cloning and protein production

#### *S. pneumoniae* PrsA and PrsA N+C

Genomic DNA from *S. pneumoniae* strain 19A was used to amplify *prsA* lacking a signal sequence (ZP_06978074.1; residues 27 to 313; primers shown in Table S2) (Fig. S14) which was cloned into the pMCSG53 expression vector containing an N-terminal 6×His-tag followed by tobacco etch virus (TEV) protease cleavage site, and encoding genes providing ampicillin resistance and rare codon tRNAs. Positive clones were verified by DNA sequencing analysis. For PrsA N+C cloning, the gblock of PrsA DNA sequence without the signal sequence and the PPIase domain was ordered from Integrated DNA Technologies. The gblock was first cloned into pCR-Blunt II-TOPO vector using the TOPO PCR Cloning Kit (Invitrogen) based on manufacturer instructions. The DNA was PCR amplified with primers *Spn*PrsASacEX_Fwd and *Spn*PrsAXmaIEX_Rev (Table S2), digested with SacI and XmaI, and gel purified. The DNA was then ligated into an N-terminal pQE30 vector (Qiagen) in frame with the 6×His-tag. Ligated reactions were transformed into Top10 *E. coli* cells. Positives clones were verified with sequencing. For protein expression for crystallization, *prsA* was expressed in *E. coli* BL21(DE3) Gold cells, grown to an OD_600_ of 0.6 at 37°C, chilled to 16°C, and induced overnight with 0.5 mM isopropyl β-d-1-thiogalactopyranoside (IPTG) at 16°C. Cells were harvested via centrifugation at 7,000 × *g*, bacterial pellets were resuspended in binding buffer (50 mM HEPES pH 7.5, 300 mM sodium chloride, 10 mM imidazole, and 2% glycerol (vol/vol) and lysed by sonication, and cell debris was removed via centrifugation at 20,000 × *g*. Cleared lysate was loaded onto a 5 mL Ni-NTA column (Qiagen) pre-equilibrated with binding buffer and extensively washed with binding buffer containing 30 mM imidazole, and protein was eluted using the above buffer supplemented with 250 mM imidazole. Fractions containing *Spn*PrsA were identified by SDS-polyacrylamide gel electrophoresis and further purified via gel filtration on a HiLoad 16/60 Superdex 75 prep-grade column equilibrated with a buffer containing 10 mM HEPES pH 7.5 and 50 mM potassium chloride. For protein production of PrsA N+C, the DNA construct was transformed into *E. coli* BL21 (DE3) cells and selected on LB agar plates containing 50 µg/mL carbenicillin. An overnight culture was used to inoculate 1 L LB broth containing 50 µg/mL of carbenicillin at 1:200 dilution. Culture sample was incubated at 37°C at 200 rpm and protein expression was induced at OD_600_ of 0.6 by the addition of 0.8 mM isopropyl β-d-1-thiogalactopyranoside. Cells were chilled to 30°C and grown for 12 hours at 200 rpm. Next, the cells were pelleted by centrifugation at 8,000 rpm for 45 min at 4°C. Resulting cell pellets were flash frozen and stored at −80°C. For protein purification, frozen cell pellets were thawed on ice and resuspended in lysis buffer (50 mM Tris, pH 7.5, 500 mM NaCl, and 25 mM imidazole; supplemented with protease inhibitor cocktail 3 [Millipore-Sigma] and DNase I [Millipore-Sigma]) at 4°C. Resuspended cells were sonicated and clarified by centrifugation at 8,000 rpm for 45 min at 4°C. Supernatants were collected and passed through an equilibrated nickel-NTA column and washed with 10 column volumes (cv) of lysis buffer. Protein was eluted with buffer E (50 mM Tris, pH 7.5, 500 mM NaCl, and 500 mM imidazole) and dialyzed overnight in the cold room in dialysis buffer (20 mM MES, pH 6.5, 100 mM NaCl, 1 mM β-mercaptoethanol, and 10% glycerol). Protein purity was determined by SDS-PAGE, and protein concentrations were determined by the bicinchoninic acid assay (Pierce).

#### *S. mutans* PrsA, *S. pyogenes* PpiA, and *S. pneumoniae* SlrA

The foldase encoded by the *prsA* gene from *S. mutans* strain UA159 lacking a signal sequence (NP_721076; residues 21–294; primers shown in Table S2) and the cyclophilin PPIase encoded by *ppiA* from *S. pyogenes* strain MGAS5005 lacking a signal sequence (AAZ50989; residues 21–268; primers shown in Table S2) were cloned using ligation-independent cloning as described previously (62, 63) into pMSCG53 vector (described above). The cyclophilin PPIase encoded by *slrA* from *S. pneumoniae* strain R6 lacking a signal sequence (ZP_06979387.1; residues 61–267; primers shown in Table S2) (Fig. S14) was cloned using ligation-independent cloning (62, 63) into pMSCG92 expression vector, which expresses protein with a C-terminal 6×His-tag preceded by a TEV protease cleavage site and encoding ampicillin resistance and genes for rare codons. This *slrA* expression construct was used for crystallization. *S. pneumoniae* strain R6 was used to clone *slrA* lacking a signal sequence (ZP_06979387.1; residues 19–267; primers shown in Table S2) into the pQE30 vector with an N-terminal 6×His-tag and encoding an ampicillin resistance gene (Qiagen). This expression construct was used for biophysical assays. Positive clones were verified by DNA sequencing analysis and proteins were expressed in *E. coli* BL21 (DE3) Magic/Star cells (64). For protein expression for crystallization, starting overnight cultures were grown in LB supplemented with 130 µg/mL ampicillin at 37°C with agitation at 220 rpm. The next day, for each clone, 3 L of M9 media (High Yield M9 SeMet media, Medicilon Inc.) supplemented with 200 µg/mL ampicillin were inoculated at 1:100 dilution with the overnight cultures and incubated at 37°C with 220 rpm agitation. Protein expression was induced at OD_600_ between 1.8 and 2 by an addition of 0.5 mM IPTG, and the cultures were further incubated at 25°C, and 200 rpm for 14 hours (64). The cells were harvested by centrifugation at 6,000 × *g* for 20 min, re-suspended in lysis buffer (10 mM Tris-HCl pH 8.3, 500 mM NaCl, 10% glycerol, 0.1% IGEPAL CA-630) (1 g of cells/5 mL of lysis buffer) and frozen at −30°C until purification.

For protein purification, frozen pellets were thawed and sonicated at 50% amplitude, in 5 s × 10 s cycle for 20 min at 4°C. The lysate was cleared by centrifugation at 18,000 × *g* for 40 min at 4°C and the supernatant was collected. The protein was purified by nickel affinity followed by size exclusion chromatography using the ÅKTAxpress system (GE Healthcare) as previously described with some modifications (65). The cell extract was loaded into a 5 mL His-Trap FF (Ni-NTA) column in loading buffer (10 mM Tris-HCl pH 8.3, 500 mM NaCl, 1 mM Tris(2-carboxyethyl) phosphine ([TCEP], and 5% glycerol), and the column was washed with 10 cv of loading buffer and 10 cv of washing buffer (10 mM Tris-HCl pH 8.3, 1 M NaCl, 25 mM imidazole, 5% glycerol). Protein was eluted with elution buffer (10 mM Tris-HCl pH 8.3, 500 mM NaCl, 1 M imidazole), loaded onto a Superdex 200 26/600 column, separated in loading buffer, collected, and analyzed by SDS-polyacrylamide gel electrophoresis. The 6×His-tag was cleaved by recombinant TEV protease at a mass ratio of 1:20 (protease:protein) overnight at room temperature. The cleaved protein was separated from recombinant TEV protease, un-cleaved protein, and the 6×His-tag peptide by Ni-NTA-affinity chromatography using loading buffer followed by loading buffer with 25 mM imidazole. The cleaved protein was collected in both in flow-through and in loading buffer with 25 mM imidazole. Both fractions were analyzed by PAGE for purity and 6×His-tag cleavage. The fraction collected in flow-through was selected; *Smu*PrsA, *Spy*PpiA, and *Spn*SlrA were concentrated to 8 mg/mL, 11 mg/mL, and 9 mg/mL, respectively, and used for crystallization.

#### *S. pneumoniae* Ply

Genomic DNA from *S. pneumoniae* strain R6 and primers listed in Table S2 were used to PCR amplify the open reading frame of *ply* (WP_001284359.1; residues 2–471) and the product was cloned into the plasmid pQE30 (Qiagen), which expresses proteins with an N-terminal 6×His-tag and encodes an ampicillin resistance gene. Positive clones were selected on LB agar plates containing 50 µg/mL carbenicillin and confirmed by DNA sequencing analysis. Ply was expressed in *E. coli* BL21 (DE3) Star cells where a single colony was picked from LB agar plates containing 50 µg/mL carbenicillin and inoculated into 10 mL of LB broth supplemented with 50 µg/mL of carbenicillin with shaking at 37°C for 16 hours. Then, 5 mL of the 16 hour culture was used to inoculate 1L LB broth containing 50 µg/mL of carbenicillin. The bacterial culture was grown until OD_600_ of 0.6, when IPTG was added to a final concentration of 0.8 mM to induce expression of the recombinant Ply protein. The bacterial culture was grown with shaking at 30°C for 12 hours and centrifuged, and pelleted cells were flash frozen and stored −80°C until further processing. Pelleted cells were thawed and resuspended in chilled wash buffer (50 mM Tris-HCl pH 7.5, 500 mM NaCl, 25 mM imidazole) and protease inhibitor cocktail set 3 (Millipore-Sigma) and DNase I (Millipore-Sigma) were added. The cells were lysed by sonication with 10 cycles of 10-seconds pulses. Lysate was centrifuged at 18,000 × *g* for 40 min, and the suspension was passed through a Ni-NTA column equilibrated with wash buffer. The protein was washed and eluted using elution buffer (50 mM Tris-HCl pH 7.5, 500 mM NaCl, 500 mM imidazole). Then, the protein was dialyzed at 4°C overnight in dialysis buffer (D) (20 mM MES, 100 mM NaCl, 1 mM β-mercaptoethanol, 10% glycerol; pH 6.5). For biophysical and biochemical assays, *S. pneumoniae prsA, prsA* N+C, *slrA*, and *S. pyogenes ppiA* were expressed and purified similarly to *ply* (Table S2). Protein concentration was measured by BCA assay (Pierce).

### Crystallization and structure determination

#### *S. pneumoniae* PrsA

Crystallization of *Spn*PrsA was performed at room temperature using the sitting drop method and 2 µL of protein or protein/ligand mixture plus 2 µL of the reservoir solution containing 2 M ammonium sulfate and 0.05 M CAPSO, pH 11. The crystals were cryoprotected in paratone oil before X-ray data collection at 100 K at the Advanced Photon Source, Argonne National Laboratory, Life Sciences Collaborative Access Team beamline (LS-CAT) 21-ID-F.

HKL3000 was used to process both Se-Met and native diffraction data sets (66). Computational corrections for absorption in a crystal and Lorentz factor were applied (67, 68). Anisotropic diffraction was corrected to adjust the error model and to compensate for the radiation-induced increase of non-isomorphism within the crystal (69–71).

The Se-Met crystal diffracted to a nominal resolution of ~2.7 Å but diffraction was highly anisotropic, with diffraction pattern extending only to ~4.0 Å resolution in the *b* direction and to a resolution better than 2.7 Å in the *a* and *c* directions. Indexing, integration, and scaling indicated P2_1_ symmetry. The diffraction from the native crystal was also anisotropic (3.2 Å in the *b* direction, ~2.45 Å in the *a* direction, and a resolution better than ~2.45 Å in the *c* direction). The native crystal had the same symmetry as the Se-Met crystal. The native data set was processed to the resolution of 2.45 Å; however, during refinement, the resolution was restricted to 2.55 Å to minimize computational instabilities resulting from highly anisotropic data.

Initial phases were obtained from Se-Met crystal in a SAD experiment. The search for heavy atom positions was performed to a resolution of 3.2 Å. The 17 Se positions were identified using SHELXD (72) with correlations coefficients: CC_All_ = 41.3%, CC_Weak_ = 25.4%, and C_FOM_ = 66.7%. Relative occupancies of Se positions varied between 0.006 and 1.000. The handedness of the best solution was determined with SHELXE. The 13 heavy atom positions were refined to 3.2 Å with MLPHARE (73), with the final figure of merit (FOM) reaching 0.345 for all observations. The density modification was performed with density maps (DM) (74–76), and included twofold non-crystallographic symmetry (NCS) averaging, with the NCS operator identified with RESOLVE (77). The resulting electron density map was used as the entry model for the model building with BUCCANEER (78) and refinement with REFMAC (79, 80), run within HKL3000 (66). The resulting main chain was ~99% complete (~1,080 aa) with ~90% side chains docked into electron density maps (~1,000 aa), and *R* factor = 35.6% and *R*_free_ = 43.1%.

The resulting assembly was used to perform isomorphous replacement with the native data set using MOLREP (81) run within HKL3000, and rebuilt and refined again with BUCCANEER and REFMAC run within HKL3000. The resulting model was then refined by iterative application of Coot and REFMAC (79, 80, 82) the local NCS restraints, and Translation–Libration–Screw (TLS) refinement (83). TLS bodies were determined with the help of the TLSMD server (84). The model quality was validated with MolProbity (85) and assessed to be satisfactory considering data resolution with the MolProbity score of 0.68, which corresponds to 100th percentile in comparison with the set of 7,646 PDB deposits solved at resolutions from 2.30 to 2.8 Å. The resolution cutoff for the refinement (2.55 Å) was selected based on visual inspection of electron density maps that was combined with refinements run with variable resolution cutoffs, where the increases of values of *R* and *R*-free in the last resolution shells were analyzed against the *R* and *R*-free values in the previous resolution shells. The final *Spn*PrsA model was deposited to the Protein Data Bank with the assigned PDB code 5TVL.

#### *S. mutans* PrsA, *S. pyogenes* PpiA, and *S. pneumoniae* SlrA

The crystallization trials for *Smu*PrsA*, Spy*PpiA, and *Spn*SlrA proteins were set up at 7.5–8 mg/mL, 10–11 mg/mL and 5–9 mg/mL, respectively, in loading buffer (10 mM Tris-HCl pH 8.3, 1 mM TCEP, and 5% glycerol) with or without 500 mM NaCl as 2 µL crystallization drops (1 µL protein:1 µL reservoir solution) in 96-well plates (Corning) using commercial Classics II, PACT, PEGs II, AmSO4, Anions, and JCSG+ (Qiagen) crystallization screens. For *Smu*PrsA, diffraction quality crystals grew from the condition with 0.1 M HEPES, pH 7.5, 20% (wt/vol) PEG 1500 (PEGs II screen, condition 18). For *Spy*PpiA, diffraction quality crystals grew in 0.2 M sodium acetate, 0.1 M Tris, pH 8.5, 30% (wt/vol) PEG 4000 (screen PEGs II, condition #46). For *Spn*SlrA, diffraction quality crystals grew in 0.2 M sodium chloride, 0.1 M MES, pH 6.0, 20% (wt/vol) PEG 6000 (screen PACT, condition #20). All crystals were cryo-cooled in liquid nitrogen for data collection.

Crystals were screened, and data were collected at the Life Sciences-Collaborative Access Team (LS-CAT) beamline 21-ID-G and 21-ID-F at the Advanced Photon Source of the Argonne National Laboratory. For *Smu*PrsA, *Spy*PpiA, and *Spn*SlrA, 400, 1,000, and 550 images were indexed, respectively. The images were integrated and scaled using HKL3000 (66). The structure of *Smu*PrsA was determined by molecular replacement with PHASER in PHENIX (86) using the structure of *Spn*PrsA (PDB 5TVL) as a search model. The structures of *Spy*PpiA and *Spn*SlrA were determined with the HKL3000 structure solution package using anomalous signal from Se-Met. All initial models went through several rounds of refinement in REFMAC v.5.8.0267 (79) and manual corrections in Coot (82). Water molecules were added using ARP/wARP software suite (74), and ligands were added to the model manually during visual inspection of electron density maps in Coot (82). TLS groups were created by the TLSMD server (84), and TLS corrections were applied during the final stages of refinement. MolProbity was used for monitoring the quality of the model during refinement and for the final validation of the structure (85). The structures were deposited to the Protein Data Bank with the assigned PDB codes 7L75 for *Smu*PrsA, 7L6Y for *Spy*PpiA, and 7L6Z for *Spn*SlrA.

### Protease-coupled PPIase assay

The PPIase assay for measuring the *cis*-to-*trans* isomerization of tetrapeptide substrates covalently linked to p-nitroanilide was previously described (47). Briefly, PPIase activity was measured using a tetrapeptide with the formula: *N*-succinyl-ala-ala-pro-phe-*p*-nitroanilide (Bachem). This tetrapeptide was dissolved in dimethyl sulfoxide to 100 µg/mL, and 100 µL per reaction was added to a 96-well flat-bottom plate on ice. Subsequently, 1 µL of 20 mg/mL chymotrypsin (Millipore-Sigma), the PPIase enzyme (10 µM final concentration), and buffer (20 mM HEPES, 140 mM NaCl, 1 mM DTT; pH 7.4) was added to a final volume of 200 µL. Then OD_390_ was measured immediately using a BioTek Cytation 5 plate reader (Agilent Technologies) over a 10 min period at 18°C–20°C. Spontaneous tetrapeptide *cis*-to-*trans* isomerization was assayed in the absence of PPIase as a negative control, and recombinant hCypA (Invitrogen) was used as a positive control. All experiments were carried out in triplicate with more than one batch of purified protein, and a minimum of three independent experiments were performed for each putative PPIase.

### Isolation of *S. pneumoniae* cell wall and supernatant proteins

The isolation of *S. pneumoniae* strain TIGR4 cell wall and supernatant fractions was described previously with minor modifications (12). Briefly, bacteria were grown to mid-log phase OD_550_ 0.4, then 10 mL total volume was normalized and fractionated. Bacterial cultures were centrifuged at 8,000 rpm for 15 min at 4°C. For assessment of Ply protein levels by Western blotting, supernatants were recovered, and trichloroacetic acid (TCA) precipitated. For assessment of Ply activity, supernatants were recovered and filtered. Cell pellets were washed with buffer containing 50 mM Tris-HCl pH 8.0 and 10 mM MgCl_2_, then resuspended in 500 µL STM buffer (0.5 M sucrose, 50 mM Tris-HCL pH 8.0, 10 mM MgCl_2_, 2 µL protease inhibitor cocktail 3, and 3 mM NaAzide) containing 100 mg/mL lysozyme and incubated for 60 min at 37°C; supernatant containing cell wall proteins were collected by centrifugation at 15,000 × *g* for 3 min at 4°C for downstream assays. For the pull-down assay, a starter culture of OD_600_ 0.2 was diluted 1:6 in a 240 mL volume and grown to OD_550_ 0.4, normalized, and fractionated for cell wall proteins as described above.

### Hemolysis assay

Ply hemolysis assays were performed as described previously with minor modifications (30). Bacterial strains were grown to mid-log phase; normalized to OD_550_ 0.4 per milliliter for a total of 10 mL for hemolytic assay, isolated cell wall fractions and supernatants were twofold serial diluted in assay buffer (1× phosphate buffered saline [PBS], pH 7.4 containing 10 mM dithiothreitol [DTT] and 0.1% bovine serum albumin) in a 96-well plate. Alsevers sheep’s RBCs were washed twice and resuspended in PBS pH 7.4. Then RBCs were diluted to 2% in assay buffer, and 50 µL was added to each 200 µL cell wall or supernatant fraction dilution and incubated at 37°C for 1 hour. For cell wall fractions, plates were centrifuged for 10 min at 233 × *g* and hemolytic units were determined upon visual inspection as the reciprocal of the highest dilution at which there was 100% lysis. For supernatant fractions, released hemoglobin from red blood cell lysis was quantified by reading the absorbance at 450 nm using the BioTek Cytation 5 plate reader (Agilent Technologies). Hemolytic units were determined as the reciprocal of the dilution resulting in 50% red blood cell lysis. Three independent *S. pneumoniae* Δ*prsA* and Δ*slrA* mutants were assayed in duplicate, and data represent three independent experiments.

### Chaperone pull-down assay and Western blot

For the pull-down assay, concentrated bacterial cell wall fractions were added into buffer Q (3.5 mM sodium phosphate, 70 mM sodium chloride, 0.01% Tween 20; pH 6.5) supplemented with protease inhibitor cocktail set 3 (Millipore-Sigma). Cell wall fractions were mixed with 800 µg of recombinant 6×His-tagged chaperones PrsA or SlrA or a no chaperone control and incubated at 4°C overnight with gentle rocking at 20 rpm. After incubation, 2 mL HisPur cobalt beads (ThermoFisher Scientific) were washed with equilibration buffer (50 mM sodium phosphate, 70 mM sodium chloride, 10 mM imidazole; pH 7.4) and the beads were added to chaperone-cell wall mixture or no chaperone control and gently rocked at 20 rpm for 2 hours at 4°C. The beads and protein mixtures were washed with four column volumes of wash buffer and eluted with buffer G (50 mM sodium phosphate, 300 mM NaCl, 150 mM imidazole; pH 7.4). Where necessary, the eluted proteins were concentrated by TCA precipitation and reconstituted in 40 µL–100 µL volume of buffer D (20 mM MES, 100 mM NaCl, 1 mM β-mercaptoethanol, 10% glycerol; pH 6.5).

For Western blotting for pull-down experiments, equal volumes of protein samples were boiled in 2× Laemmli sample buffer for 10 min and run on a 10% SDS-PAGE gel at 150 V for 1 hour. Protein bands were transferred onto a polyvinylidene difluoride membranes (Millipore) at 23 V for 90 min and blocked overnight with either 1× PBS with 0.05% Tween-20 and 5% non-fat dry milk or in TBS-0.05% Tween-20 (20 mM Tris, 500 mM NaCl; pH 7.4) and BSA at 4°C overnight. Next, sample membranes were washed three times and probed with polyclonal anti-PrsA (1:2,000 dilution), anti-SlrA (1:1,000 dilution), and monoclonal Ply (1:1,000 dilution) primary antibodies for 1 hour at room temperature. Membranes were further washed three times and probed with secondary antibodies. PrsA and SlrA were probed with a secondary antibody conjugated to alkaline phosphatase at 1:5,000 and 1:2,500 dilutions, respectively. Ply was probed with either horseradish peroxidase or alkaline phosphatase-conjugated secondary antibody at 1:5,000 dilution for 1 hour. Protein bands were acquired using 1-step NBT/BCIP (5-bromo-4-chloro-3-indolyl-phosphate–nitroblue tetrazolium) ready-made substrate solution (ThermoFisher Scientific) or by the ChemiDoc Imaging Systems (Bio-Rad Laboratories, Hercules, CA). ImageJ software (NIH) was used for densitometry and all protein amounts were compared to the wild-type set at 100% for cell wall and supernatant samples. Data were represented as mean ± standard deviations.

### DSF

DSF was performed using the QuantStudio 3 Real-Time PCR System (Applied Biosystems) as previously described (87). Briefly, reaction mixtures were diluted in 96-well Real-Time PCR Plates (Eppendorf), and each single reaction contained protein samples at a 2.5 µM final concentration and 5× SYPRO Orange (ThermoFisher Scientific) in 1× PBS buffer, pH 7.4. Triplicates of samples were diluted in volumes of 20 µL, sealed, and centrifuged at 1,800 rpm for 2 min before loading. Sample temperatures were ramped at ~1% (1.6°C per minute) from 30°C to 95°C. Data were exported into the ThermoFisher cloud and analyzed. Melting temperatures (*T*_*m*_) were determined from the derivative of the fluorescence intensity with respect to the temperature.

### CD spectroscopy

CD spectroscopy data were collected using the JASCO J-1500 machine at 25°C using the 0.2 mm path length cuvette from 260 nm to 190 nm range as described previously (31). Recombinant protein samples were diluted to 10 µM using 20 mM KH_2_PO_4_ buffer, pH 7.4. Three spectra scans each were collected for all samples and averaged using the Spectra Manager, and data were analyzed using the Spectra Analysis Software v.1.07.04 (Build 2). A figure was rendered using the GraphPad Prism (v.10.2.3).

### Microscale thermophoresis and data analysis

Proteins were labeled with NanoTemper 2nd Generation Red NHS Dye (Nanotemper Technologies), photosensitive samples were incubated for 30 min in a dark room, and then passed through a Nanotemper B-column to remove excess red NHS dye, and the flow-through containing each labeled protein was collected. The degree of label (DOL) was calculated using the formula: A_650_/195,000 /M/cm × concentration of labeled protein, where A_650_ represents absorbance at wavelength 650 nm and 195,000 /M/cm represents the molar absorbance co-efficient of the red NHS dye. A DOL value of 0.7–1.0 was considered optimal labeling. Both the labeled and the unlabeled proteins were maintained at pH 6.5. The labeled proteins were aliquoted into small volumes, flash frozen, and stored at −80°C. To test the binding interaction between two proteins (labeled and unlabeled), a serial dilution using 10 µL of the unlabeled protein was prepared in low-binding microfuge tubes and 10 µL of labeled protein (20 nM final concentration) was added to each serial dilution. Then, 10 µL of each reaction was loaded into a capillary tube (Nanotemper Technologies) where each sample contained 0.05% Τween 20 to prevent aggregation. The capillary tubes were loaded into the Monolith MST machine and ran at 20%–100% excitation power and 50% MST Power at 25°C. Data were analyzed using PALMIST Analysis Software (50) and rendered with GUSSI (51). For analysis, raw data were imported into PALMIST, a 1:1 *K*_*d*_ fitting parameter was applied with 95% CI, the resulting dissociation constants are shown as *K*_*d*_ (lower limit, upper limit). For figure generation, analyzed data were rendered in GUSSI, the top panel shows the normalized fluorescence, the middle panel shows the data curve, and the bottom fit shows the residuals of a plot between the fitted line and the data. The standard deviation of three or more independent replicates from at least two purified protein batches was plotted.

### ITC

Frozen purified protein aliquots were thawed and then degassed for 15 min while acclimating to room temperature (25°C). The ITC sample cell was equilibrated three times with 500 µL of storage buffer (20 mM MES, 100 mM NaCl, 1 mM β-mercaptoethanol, 10% glycerol; pH 6.5) before protein loading. The loading syringe was equilibrated with 300 µL storage buffer before protein was loaded. After heat levels were stabilized, volumes between 2 and 5 µL of protein in the syringe per step were injected into the sample cell 20 times, with 300 to 400 seconds between each injection. The raw heat rates were converted to binding enthalpies (kJ/mol) and the heat of dilution from control experiments was used as a correction factor. The independent binding model was used for the non-linear regression curve fit using NanoAnalyze software (TA instruments).

### Chaperone-assisted protein folding assay

Purified Ply (0.2 µM) was denatured in buffer C (8 M urea, 30 mM MOPS-HCl, 1 mM beta-mercaptoethanol; pH 6.5) (88) for 1 hour at room temperature. For the folding assay, the denatured Ply was diluted 1:20 using ice-cold buffer F (137 mM NaCl, 2.7 mM KCl, 10 mM Na_2_HPO_4_/KH_2_PO_4_; pH 6.5) supplemented with 1 mM DTT. Folding of denatured Ply was initiated by the addition of a known concentration of chaperone (*Spn*PrsA or *Spn*SlrA) at 25°C for 5 min. Next, the hemolytic activity of functional Ply was determined with the addition of 25% Alsevers sheep’s RBCs followed by incubation for 45 min at room temperature (89). After incubation, the RBCs were pelleted by centrifugation at 1,800 rpm for 5 min at 4°C. The amount of hemoglobin released from RBC lysis was determined by measuring the absorbance at 450 nm using the BioTek Cytation 5 plate reader (Agilent Technologies). For a 100% RBC lysis control, RBCs were lysed with 100 µL water or 1% Triton X-100. The hemolytic activity was calculated as the reciprocal of the dilution at which 50% of the RBCs were lysed. The hemolytic rates of denatured Ply in the presence of the chaperones were performed similarly with few modifications. Briefly, urea-denatured Ply was diluted with assay buffer 20-fold and incubated in the presence of the chaperones for 5 min. The hemolytic activity of functional Ply was then measured after the addition of Alsevers sheep’s RBCs and incubation from 0 to 95 min. Two independent experiments with three replicates each were performed using denatured Ply.

### Statistical analyses

Data and statistical analyses were performed using the Prism software v.10.2.3 (GraphPad Software, Inc.). Unless otherwise noted, PyMol v.2.5.4 (90) (Schrödinger, LLC) was used for figure preparation.

## Data Availability

All data are included in this article or in the supporting information. X-ray crystal structures are deposited in the Protein Data Bank (PDB) under the following accession codes: *Spn*PrsA (5TVL), *Smu*PrsA (7L75), *Spn*SlrA (7l6Z), and *Spy*PpiA (7l6Y).

## SUPPLEMENTAL MATERIAL

Supporting Information msphere.01078-24-s0001.pdf Supplemental figures and tables.

ASM does not own the copyrights to Supplemental Material that may be linked to, or accessed through, an article. The authors have granted ASM a non-exclusive, world-wide license to publish the Supplemental Material files. Please contact the corresponding author directly for reuse.

## References

[1] Saio T, Guan X, Rossi P, Economou A, Kalodimos CG. 2014. Structural basis for protein antiaggregation activity of the trigger factor chaperone. Science 344:1250494. doi:10.1126/science.1250494 24812405 PMC4070327

[2] Sarvas M, Harwood CR, Bron S, Dijl JM. 2004. Post-translocational folding of secretory proteins in Gram-positive bacteria, p 311–327. Elsevier. 10.1016/j.bbamcr.2004.04.009 15546674

[3] Mitra R, Wu K, Lee C, Bardwell JCA. 2022. ATP-independent chaperones. Annu Rev Biophys 51:409–429. doi:10.1146/annurev-biophys-090121-082906 35167761

[4] Stull F, Koldewey P, Humes JR, Radford SE, Bardwell JCA. 2016. Substrate protein folds while it is bound to the ATP-independent chaperone Spy. Nat Struct Mol Biol 23:53–58. doi:10.1038/nsmb.3133 26619265 PMC4847750

[5] Henderson B, Allan E, Coates ARM. 2006. Stress wars: the direct role of host and bacterial molecular chaperones in bacterial infection. Infect Immun 74:3693–3706. doi:10.1128/IAI.01882-05 16790742 PMC1489680

[6] Kadioglu A, Weiser JN, Paton JC, Andrew PW. 2008. The role of *Streptococcus pneumoniae* virulence factors in host respiratory colonization and disease, p 288–301. Nature Publishing Group. 10.1038/nrmicro1871 18340341

[7] Kreikemeyer B, McIver KS, Podbielski A. 2003. Virulence factor regulation and regulatory networks in *Streptococcus pyogenes* and their impact on pathogen-host interactions, p 224–232. Elsevier Current Trends. 10.1016/s0966-842x(03)00098-2 12781526

[8] Spellerberg B, Brandt C. 2015. *Streptococcus*. Manual of clinical microbiology. doi:10.1128/9781555817381.ch22:383-402

[9] Nguyen CT, Park SS, Rhee DK. 2015. Stress responses in *Streptococcus* species and their effects on the host, p 741–749. Springer. 10.1007/s12275-015-5432-6 26502957

[10] Mattoo RUH, Goloubinoff P. 2014. Molecular chaperones are nanomachines that catalytically unfold misfolded and alternatively folded proteins. Cell Mol Life Sci 71:3311–3325. doi:10.1007/s00018-014-1627-y 24760129 PMC4131146

[11] Saio T, Kawagoe S, Ishimori K, Kalodimos CG. 2018. Oligomerization of a molecular chaperone modulates its activity. Elife 7:e35731. doi:10.7554/eLife.35731 29714686 PMC5988418

[12] George JL, Agbavor C, Cabo LF, Cahoon LA. 2024. Streptococcus pneumoniae secretion chaperones PrsA, SlrA, and HtrA are required for competence, antibiotic resistance, colonization, and invasive disease. Infect Immun 92:e0049023. doi:10.1128/iai.00490-23 38226817 PMC10863415

[13] Cahoon LA, Freitag NE, Prehna G. 2016. A structural comparison of Listeria monocytogenes protein chaperones PrsA1 and PrsA2 reveals molecular features required for virulence. Mol Microbiol 101:42–61. doi:10.1111/mmi.13367 27007641 PMC4925323

[14] Wu ZY, Campeau A, Liu CH, Gonzalez DJ, Yamaguchi M, Kawabata S, Lu CH, Lai CY, Chiu HC, Chang YC. 2021. Unique virulence role of post-translocational chaperone PrsA in shaping Streptococcus pyogenes secretome. Virulence 12:2633–2647. doi:10.1080/21505594.2021.1982501 34592883 PMC8489961

[15] Hermans PWM, Adrian PV, Albert C, Estevão S, Hoogenboezem T, Luijendijk IHT, Kamphausen T, Hammerschmidt S. 2006. The streptococcal lipoprotein rotamase A (SlrA) is a functional peptidyl-prolyl isomerase involved in pneumococcal colonization. J Biol Chem 281:968–976. doi:10.1074/jbc.M510014200 16260779

[16] Shaw PE. 2002. Peptidyl-prolyl isomerases: a new twist to transcription. EMBO Rep 3:521–526. doi:10.1093/embo-reports/kvf118 12052773 PMC1084152

[17] Mukouhara T, Arimoto T, Cho K, Yamamoto M, Igarashi T. 2011. Surface lipoprotein PpiA of Streptococcus mutans suppresses scavenger receptor MARCO-dependent phagocytosis by macrophages. Infect Immun 79:4933–4940. doi:10.1128/IAI.05693-11 21986627 PMC3232644

[18] Göthel SF, Marahiel MA. 1999. Peptidyl-prolyl cis-trans isomerases, a superfamily of ubiquitous folding catalysts. Cell Mol Life Sci 55:423–436. doi:10.1007/s000180050299 10228556 PMC11146858

[19] Ünal CM, Steinert M. 2014. Microbial peptidyl-prolyl cis/trans isomerases (PPIases): virulence factors and potential alternative drug targets. Microbiol Mol Biol Rev 78:544–571. doi:10.1128/MMBR.00015-14 25184565 PMC4187684

[20] Guo L, Wu T, Hu W, He X, Sharma S, Webster P, Gimzewski JK, Zhou X, Lux R, Shi W. 2013. Phenotypic characterization of the foldase homologue PrsA in Streptococcus mutans. Mol Oral Microbiol 28:154–165. doi:10.1111/omi.12014 23241367 PMC3819222

[21] Stamnes MA, Rutherford SL, Zuker CS. 1992. Cyclophilins: a new family of proteins involved in intracellular folding. Trends Cell Biol 2:272–276. doi:10.1016/0962-8924(92)90200-7 14731520

[22] Cron LE, Bootsma HJ, Noske N, Burghout P, Hammerschmidt S, Hermans PWM. 2009. Surface-associated lipoprotein PpmA of Streptococcus pneumoniae is involved in colonization in a strain-specific manner. Microbiology (Reading) 155:2401–2410. doi:10.1099/mic.0.026765-0 19389773

[23] Overweg K, Kerr A, Sluijter M, Jackson MH, Mitchell TJ, de Jong AP, de Groot R, Hermans PW. 2000. The putative proteinase maturation protein A of Streptococcus pneumoniae is a conserved surface protein with potential to elicit protective immune responses. Infect Immun 68:4180–4188. doi:10.1128/IAI.68.7.4180-4188.2000 10858235 PMC101721

[24] Cahoon LA, Freitag NE. 2015. Identification of conserved and species-specific functions of the Listeria monocytogenes PrsA2 secretion chaperone. Infect Immun 83:4028–4041. doi:10.1128/IAI.00504-15 26216425 PMC4567654

[25] Hsu CC, Hsu RB, Oon XH, Chen YT, Chen JW, Hsu CH, Kuo YM, Shih YH, Chia JS, Jung CJ. 2022. Streptococcus mutans PrsA mediates AtlA secretion contributing to extracellular DNA release and biofilm formation in the pathogenesis of infective endocarditis. Virulence 13:1379–1392. doi:10.1080/21505594.2022.2105351 35876630 PMC9377233

[26] Crowley PJ, Brady LJ. 2016. Evaluation of the effects of Streptococcus mutans chaperones and protein secretion machinery components on cell surface protein biogenesis, competence, and mutacin production. Mol Oral Microbiol 31:59–77. doi:10.1111/omi.12130 26386361 PMC4712094

[27] Nishimoto AT, Rosch JW, Tuomanen EI. 2020. Pneumolysin: pathogenesis and therapeutic target. Front Microbiol 11:1543–1543. doi:10.3389/fmicb.2020.01543 32714314 PMC7343714

[28] Rubins JB, Charboneau D, Paton JC, Mitchell TJ, Andrew PW, Janoff EN. 1995. Dual function of pneumolysin in the early pathogenesis of murine pneumococcal pneumonia. J Clin Invest 95:142–150. doi:10.1172/JCI117631 7814608 PMC295392

[29] Price KE, Camilli A. 2009. Pneumolysin localizes to the cell wall of Streptococcus pneumoniae. J Bacteriol 191:2163–2168. doi:10.1128/JB.01489-08 19168620 PMC2655535

[30] Price KE, Greene NG, Camilli A. 2012. Export requirements of pneumolysin in Streptococcus pneumoniae. J Bacteriol 194:3651–3660. doi:10.1128/JB.00114-12 22563048 PMC3393478

[31] Agbavor C, Zimnicka A, Kumar A, George JL, Torres M, Prehna G, Alonzo F 3rd, Durrant JD, Freitag NE, Cahoon LA. 2024. The chaperone PrsA2 regulates the secretion, stability, and folding of listeriolysin O during Listeria monocytogenes infection. MBio 15:e0074324. doi:10.1128/mbio.00743-24 38809022 PMC11253611

[32] Alonzo F III, Port GC, Cao M, Freitag NE. 2009. The posttranslocation chaperone PrsA2 contributes to multiple facets of Listeria monocytogenes pathogenesis . Infect Immun 77:2612–2623. doi:10.1128/IAI.00280-09 19451247 PMC2708592

[33] Krissinel E, Henrick K. 2007. Inference of macromolecular assemblies from crystalline state. J Mol Biol 372:774–797. doi:10.1016/j.jmb.2007.05.022 17681537

[34] Li Z, Jaroszewski L, Iyer M, Sedova M, Godzik A. 2020. FATCAT 2.0: towards a better understanding of the structural diversity of proteins. Nucleic Acids Res 48:W60–W64. doi:10.1093/nar/gkaa443 32469061 PMC7319568

[35] Jakob RP, Koch JR, Burmann BM, Schmidpeter PAM, Hunkeler M, Hiller S, Schmid FX, Maier T. 2015. Dimeric structure of the bacterial extracellular foldase PrsA. J Biol Chem 290:3278–3292. doi:10.1074/jbc.M114.622910 25525259 PMC4319002

[36] Jones P, Binns D, Chang HY, Fraser M, Li W, McAnulla C, McWilliam H, Maslen J, Mitchell A, Nuka G, Pesseat S, Quinn AF, Sangrador-Vegas A, Scheremetjew M, Yong SY, Lopez R, Hunter S. 2014. InterProScan 5: genome-scale protein function classification. Bioinformatics 30:1236–1240. doi:10.1093/bioinformatics/btu031 24451626 PMC3998142

[37] Pribyl T, Moche M, Dreisbach A, Bijlsma JJE, Saleh M, Abdullah MR, Hecker M, van Dijl JM, Becher D, Hammerschmidt S. 2014. Influence of impaired lipoprotein biogenesis on surface and exoproteome of Streptococcus pneumoniae. J Proteome Res 13:650–667. doi:10.1021/pr400768v 24387739

[38] Pei J, Kim BH, Grishin NV. 2008. PROMALS3D: a tool for multiple protein sequence and structure alignments. Nucleic Acids Res 36:2295–2300. doi:10.1093/nar/gkn072 18287115 PMC2367709

[39] Robert X, Gouet P. 2014. Deciphering key features in protein structures with the new ENDscript server. Nucleic Acids Res 42:W320–W324. doi:10.1093/nar/gku316 24753421 PMC4086106

[40] Alonzo III F, Xayarath B, Whisstock JC, Freitag NE. 2011. Functional analysis of the Listeria monocytogenes secretion chaperone PrsA2 and its multiple contributions to bacterial virulence . Mol Microbiol 80:1530–1548. doi:10.1111/j.1365-2958.2011.07665.x 21545417 PMC3115453

[41] Holm L. 2020. Using Dali for protein structure comparison. Methods Mol Biol 2112:29–42. doi:10.1007/978-1-0716-0270-6_3 32006276

[42] Davis TL, Walker JR, Campagna-Slater V, Finerty PJ, Paramanathan R, Bernstein G, MacKenzie F, Tempel W, Ouyang H, Lee WH, Eisenmesser EZ, Dhe-Paganon S. 2010. Structural and biochemical characterization of the human cyclophilin family of peptidyl-prolyl isomerases. PLoS Biol 8:e1000439. doi:10.1371/journal.pbio.1000439 20676357 PMC2911226

[43] Sievers F, Wilm A, Dineen D, Gibson TJ, Karplus K, Li W, Lopez R, McWilliam H, Remmert M, Söding J, Thompson JD, Higgins DG. 2011. Fast, scalable generation of high-quality protein multiple sequence alignments using Clustal Omega. Mol Syst Biol 7:539. doi:10.1038/msb.2011.75 21988835 PMC3261699

[44] Madej T, Marchler-Bauer A, Lanczycki C, Zhang D, Bryant SH. 2020. Biological assembly comparison with VAST. Methods Mol Biol 2112:175–186. doi:10.1007/978-1-0716-0270-6_13 32006286

[45] Madej T, Lanczycki CJ, Zhang D, Thiessen PA, Geer RC, Marchler-Bauer A, Bryant SH. 2014. MMDB and VAST+: tracking structural similarities between macromolecular complexes. Nucleic Acids Res 42:D297–D303. doi:10.1093/nar/gkt1208 24319143 PMC3965051

[46] Abramson J, Adler J, Dunger J, Evans R, Green T, Pritzel A, Ronneberger O, Willmore L, Ballard AJ, Bambrick J, et al.. 2024. Accurate structure prediction of biomolecular interactions with AlphaFold 3. Nature New Biol 630:493–500. doi:10.1038/s41586-024-07487-w PMC11168924 38718835

[47] Fischer G, Wittmann-Liebold B, Lang K, Kiefhaber T, Schmid FX. 1989. Cyclophilin and peptidyl-prolyl cis-trans isomerase are probably identical proteins. Nature New Biol 337:476–478. doi:10.1038/337476a0 2492638

[48] Handschumacher RE, Harding MW, Rice J, Drugge RJ, Speicher DW. 1984. Cyclophilin: a specific cytosolic binding protein for cyclosporin A. Science 226:544–547. doi:10.1126/science.6238408 6238408

[49] Alonzo F III, Freitag NE. 2010. Listeria monocytogenes PrsA2 is required for virulence factor secretion and bacterial viability within the host cell cytosol . Infect Immun 78:4944–4957. doi:10.1128/IAI.00532-10 20823208 PMC2976329

[50] Scheuermann TH, Padrick SB, Gardner KH, Brautigam CA. 2016. On the acquisition and analysis of microscale thermophoresis data. Anal Biochem 496:79–93. doi:10.1016/j.ab.2015.12.013 26739938 PMC4873313

[51] Brautigam CA. 2015. Calculations and publication-quality illustrations for analytical ultracentrifugation data, p 109–133. In Methods in enzymology. Elsevier. 10.1016/bs.mie.2015.05.001 26412649

[52] Lawrence SL, Feil SC, Morton CJ, Farrand AJ, Mulhern TD, Gorman MA, Wade KR, Tweten RK, Parker MW. 2015. Crystal structure of Streptococcus pneumoniae pneumolysin provides key insights into early steps of pore formation. Sci Rep 5:14352. doi:10.1038/srep14352 26403197 PMC4585913

[53] Sučec I, Bersch B, Schanda P. 2021. How do chaperones bind (partly) unfolded client proteins? Front Mol Biosci 8:1017–1017. doi:10.3389/fmolb.2021.762005 PMC8573040 34760928

[54] Bose D, Chakrabarti A. 2017. Substrate specificity in the context of molecular chaperones. IUBMB Life 69:647–659. doi:10.1002/iub.1656 28748601

[55] Bandara M, Skehel JM, Kadioglu A, Collinson I, Nobbs AH, Blocker AJ, Jenkinson HF. 2017. The accessory Sec system (SecY2A2) in Streptococcus pneumoniae is involved in export of pneumolysin toxin, adhesion and biofilm formation. Microbes Infect 19:402–412. doi:10.1016/j.micinf.2017.04.003 28456649 PMC5526788

[56] Tilley SJ, Orlova EV, Gilbert RJC, Andrew PW, Saibil HR. 2005. Structural basis of pore formation by the bacterial toxin pneumolysin. Cell 121:247–256. doi:10.1016/j.cell.2005.02.033 15851031

[57] Cahoon LA, Freitag NE. 2014. Listeria monocytogenes virulence factor secretion: don’t leave the cell without a chaperone. Front Cell Infect Microbiol 4:13. doi:10.3389/fcimb.2014.00013 24575392 PMC3921577

[58] Cahoon LA, Alejandro-Navarreto X, Gururaja AN, Light SH, Alonzo F, Anderson WF, Freitag NE. 2022. Listeria monocytogenes two component system PieRS regulates secretion chaperones PrsA1 and PrsA2 and enhances bacterial translocation across the intestine. Mol Microbiol 118:278–293. doi:10.1111/mmi.14967 35943959 PMC9545042

[59] Scheuplein NJ, Bzdyl NM, Kibble EA, Lohr T, Holzgrabe U, Sarkar-Tyson M. 2020. Targeting protein folding: a novel approach for the treatment of pathogenic bacteria, p 13355–13388. American Chemical Society. 10.1021/acs.jmedchem.0c00911 32786507

[60] Bergmann S, Hammerschmidt S. 2006. Versatility of pneumococcal surface proteins, p 295–303. Microbiology Society. 10.1099/mic.0.28610-0 16436417

[61] Kaufmann SH, Hess J. 1999. Impact of intracellular location of and antigen display by intracellular bacteria: implications for vaccine development. Immunol Lett 65:81–84. doi:10.1016/s0165-2478(98)00128-x 10065631

[62] Stols L, Gu M, Dieckman L, Raffen R, Collart FR, Donnelly MI. 2002. A new vector for high-throughput, ligation-independent cloning encoding a tobacco etch virus protease cleavage site. Protein Expr Purif 25:8–15. doi:10.1006/prep.2001.1603 12071693

[63] Kim Y, Bigelow L, Borovilos M, Dementieva I, Duggan E, Eschenfeldt W, Hatzos C, Joachimiak G, Li H, Maltseva N, Mulligan R, Quartey P, Sather A, Stols L, Volkart L, Wu R, Zhou M, Joachimiak A. 2008. Chapter 3. High-throughput protein purification for x-ray crystallography and NMR. Adv Protein Chem Struct Biol 75:85–105. doi:10.1016/S0065-3233(07)75003-9 20731990 PMC3366499

[64] Kwon K, Peterson SN. 2014. High-throughput cloning for biophysical applications, p 61–74. In Structural genomics and drug discovery. Springer. 10.1007/978-1-4939-0354-2_5 24590709

[65] Shuvalova L. 2014. Parallel protein purification, p 137–143. In Structural genomics and drug discovery. Springer. 10.1007/978-1-4939-0354-2_10 24590714

[66] Minor W, Cymborowski M, Otwinowski Z, Chruszcz M. 2006. HKL-3000: the integration of data reduction and structure solution--from diffraction images to an initial model in minutes. Acta Crystallogr D Biol Crystallogr 62:859–866. doi:10.1107/S0907444906019949 16855301

[67] Egelstaff PA, Eder OJ, Glaser W, Polo J, Renker B, Soper AK. 1990. Dynamic-structure-factor measurements on a model Lorentz gas. Phys Rev A 41:1936–1942. doi:10.1103/physreva.41.1936 9903304

[68] Lackner T, Posch M. 1987. Dynamical self-structure factor Ss(q, ω) for inverse-power-law interactions in the Rayleigh and Lorentz limits. Phys Rev A Gen Phys 36:5401–5414. doi:10.1103/physreva.36.5401 9898810

[69] Otwinowski Z, Borek D, Majewski W, Minor W. 2003. Multiparametric scaling of diffraction intensities. Acta Crystallogr A Found Crystallogr 59:228–234. doi:10.1107/S0108767303005488 12714773

[70] Borek D, Cymborowski M, Machius M, Minor W, Otwinowski Z. 2010. Diffraction data analysis in the presence of radiation damage. Acta Crystallogr D Biol Crystallogr 66:426–436. doi:10.1107/S0907444909040177 20382996 PMC2852307

[71] Borek D, Dauter Z, Otwinowski Z. 2013. Identification of patterns in diffraction intensities affected by radiation exposure. J Synchrotron Radiat 20:37–48. doi:10.1107/S0909049512048807 23254654 PMC3526920

[72] Dall’Antonia F, Baker PJ, Schneider TR. 2003. Optimization of selenium substructures as obtained from SHELXD. Acta Crystallogr D Biol Crystallogr 59:1987–1994. doi:10.1107/s0907444903017670 14573954

[73] Dodson EJ, Winn M, Ralph A. 1997. Collaborative computational project, number 4: providing programs for protein crystallography. Methods Enzymol 277:620–633. doi:10.1016/s0076-6879(97)77034-4 18488327

[74] Morris RJ, Perrakis A, Lamzin VS. 2003. ARP/wARP and automatic interpretation of protein electron density maps, p 229–244. In Methods in enzymology. Elsevier. 10.1016/S0076-6879(03)74011-7 14696376

[75] Terwilliger TC. 2000. Maximum-likelihood density modification. Acta Crystallogr D Biol Crystallogr 56:965–972. doi:10.1107/s0907444900005072 10944333 PMC2792768

[76] Terwilliger TC. 2001. Maximum-likelihood density modification using pattern recognition of structural motifs. Acta Crystallogr D Biol Crystallogr 57:1755–1762. doi:10.1107/s0907444901013737 11717487 PMC2745886

[77] Terwilliger TC. 2003. Automated main-chain model building by template matching and iterative fragment extension. Acta Crystallogr D Biol Crystallogr 59:38–44. doi:10.1107/s0907444902018036 12499537 PMC2745878

[78] Cowtan K. 2006. The Buccaneer software for automated model building. 1. Tracing protein chains. Acta Crystallogr D Biol Crystallogr 62:1002–1011. doi:10.1107/S0907444906022116 16929101

[79] Murshudov GN, Skubák P, Lebedev AA, Pannu NS, Steiner RA, Nicholls RA, Winn MD, Long F, Vagin AA. 2011. REFMAC5 for the refinement of macromolecular crystal structures. Acta Crystallogr D Biol Crystallogr 67:355–367. doi:10.1107/S0907444911001314 21460454 PMC3069751

[80] Winn MD, Murshudov GN, Papiz MZ. 2003. Macromolecular TLS refinement in REFMAC at moderate resolutions, p 300–321. In Methods in enzymology. Elsevier. 10.1016/S0076-6879(03)74014-2 14696379

[81] Lebedev AA, Vagin AA, Murshudov GN. 2008. Model preparation in MOLREP and examples of model improvement using X-ray data. Acta Crystallogr D Biol Crystallogr 64:33–39. doi:10.1107/S0907444907049839 18094465 PMC2394799

[82] Emsley P, Cowtan K. 2004. Coot: model-building tools for molecular graphics. Acta Crystallogr D Biol Crystallogr 60:2126–2132. doi:10.1107/S0907444904019158 15572765

[83] Painter J, Merritt EA. 2006. Optimal description of a protein structure in terms of multiple groups undergoing TLS motion. Acta Crystallogr D Biol Crystallogr 62:439–450. doi:10.1107/S0907444906005270 16552146

[84] Painter J, Merritt EA. 2006. TLSMD web server for the generation of multi-group TLS models. J Appl Crystallogr 39:109–111. doi:10.1107/S0021889805038987

[85] Chen VB, Arendall WB III, Headd JJ, Keedy DA, Immormino RM, Kapral GJ, Murray LW, Richardson JS, Richardson DC. 2010. MolProbity: all-atom structure validation for macromolecular crystallography . Acta Crystallogr D Biol Crystallogr 66:12–21. doi:10.1107/S0907444909042073 20057044 PMC2803126

[86] Adams PD, Afonine PV, Bunkóczi G, Chen VB, Davis IW, Echols N, Headd JJ, Hung L-W, Kapral GJ, Grosse-Kunstleve RW, McCoy AJ, Moriarty NW, Oeffner R, Read RJ, Richardson DC, Richardson JS, Terwilliger TC, Zwart PH. 2010. PHENIX: a comprehensive Python-based system for macromolecular structure solution. Acta Crystallogr D Biol Crystallogr 66:213–221. doi:10.1107/S0907444909052925 20124702 PMC2815670

[87] Ehrhardt MKG, Warring SL, Gerth ML. 2018. Screening chemoreceptor–ligand interactions by high-throughput thermal-shift assays. Methods Mol Biol 1729:281–290. doi:10.1007/978-1-4939-7577-8_22 29429098

[88] Grimsley GR, Huyghues-Despointes BMP, Pace CN, Scholtz JM. 2006. Preparation of urea and guanidinium chloride stock solutions for measuring denaturant-induced unfolding curves. CSH Protoc 2006:rot4241. doi:10.1101/pdb.prot4241 22485638

[89] Köster S, van Pee K, Hudel M, Leustik M, Rhinow D, Kühlbrandt W, Chakraborty T, Yildiz Ö. 2014. Crystal structure of listeriolysin O reveals molecular details of oligomerization and pore formation. Nat Commun 5:3690. doi:10.1038/ncomms4690 24751541

[90] DeLano WL. 2002. The PyMOL molecular graphics system. Available from: https://www.pymol.org/

