# Supplementary material for: Structural analysis of extracellular ATP-independent chaperones of streptococcal species and protein substrate interactions
Source: mSphere. 2025 Jan 29;10(2):e01078-24. doi: 10.1128/msphere.01078-24 (PMC11853100; doi:10.1128/msphere.01078-24)
Supplement: Supporting Information — Supplemental figures and tables. [file msphere.01078-24-s0001.pdf]

Supporting Information for

Structural analysis of secreted ATP-independent chaperones of Streptococcal species and protein substrate interactions

Charles Agbavor[1], Madeline Torres[1], Nicole L. Inniss[2,3], Sarah E. Latimer[1],George Minasov[2,3], Ludmilla Shuvalova[4], Zdzislaw Wawrzak[5], Dominika Borek[3,6,7], Zbyszek Otwinowski[6,7], Peter J. Stogios[8], Alexei Savchenko[3,8,9], Wayne F. Anderson[10], Karla J. F. Satchell[2,3], Laty A. Cahoon[1*]

*Corresponding author
latycahoon@pitt.edu

This PDF file includes:

Supplemental Figures

S1: Structure of *S. pneumoniae* PrsA tetramer (PDB 5TVL).
S2: Structural comparison of *S. pneumoniae* PrsA and *S. mutans* PrsA dimers.
S3: The C-terminal tail of PrsA proteins are intrinsically disordered and enriched with serine residues.
S4: Structural comparison *S. pneumoniae* SlrA and *S. pyogenes* PpiA PPIase structures.
S5: The asymmetric unit content of *S. pneumoniae* SlrA (PDB 7L6Z).
S6: SlrA-like appendages may function as domains for substrate recognition or activity.
S7: The pneumolysin is significantly reduced in the cell wall fraction of *prsA* and *slrA* mutants.
S8: PrsA and SlrA are required for the secretion of functional pneumolysin.
S9: The PPIase domain of *S. pneumoniae* PrsA is required for efficient binding to the pneumolysin.
S10: Thermal stability and circular dichroism spectroscopy (CD) analysis of PrsA and PrsA N+C proteins.
S11: PrsA and SlrA possess residual binding for bovine serum albumin (BSA) and lysozyme.
S12: *S. pneumoniae* PrsA and SlrA possess inhibitory protein folding/holdase activity at high chaperone concentrations.
S13: Specificity of *S. pneumoniae* PrsA and SlrA chaperones.
S14: Conservation of PrsA and SlrA in *S. pneumoniae* strains used in this study.
S15: SDS-PAGE of purified recombinant proteins.

Supplemental Tables

S1: List of bacterial strains and plasmids.
S2: List of oligonucleotides.

Figure S1

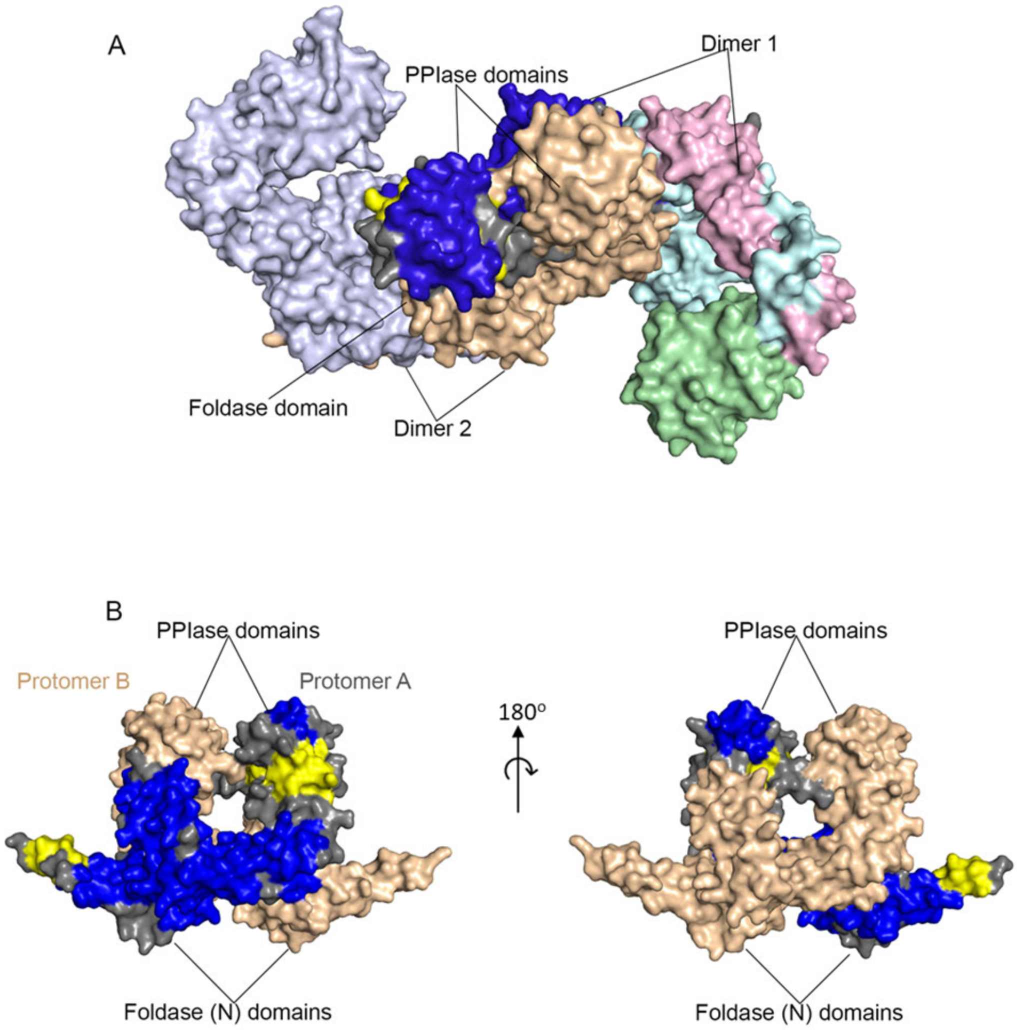

Figure S1: Structure of *S. pneumoniae* PrsA tetramer (PDB 5TVL). A) Surface representation of *Spn*PrsA (PDB 5TVL) shows Dimer 1 (chains A and D) interacting with Dimer 2 (chains B and C). In chain A, regions composed of α-helices are shown in blue, β-strands are shown in yellow, and loops are shown in grey, chain D is colored by domain (PPIase, pale green; N- and C-terminal regions of the foldase domain are pale blue and pale pink, respectively), chain B is wheat, and chain C is light violet. A PPIase domain of each dimer is seen in the hydrophobic pocket of the other dimer. B) A focused view of the surface representation showing the interaction between chains A and B. The image on the right is rotated180º about the y-axis with respect to the image on the left. The interface was calculated using the PISAPDB Server (1).

Figure S2

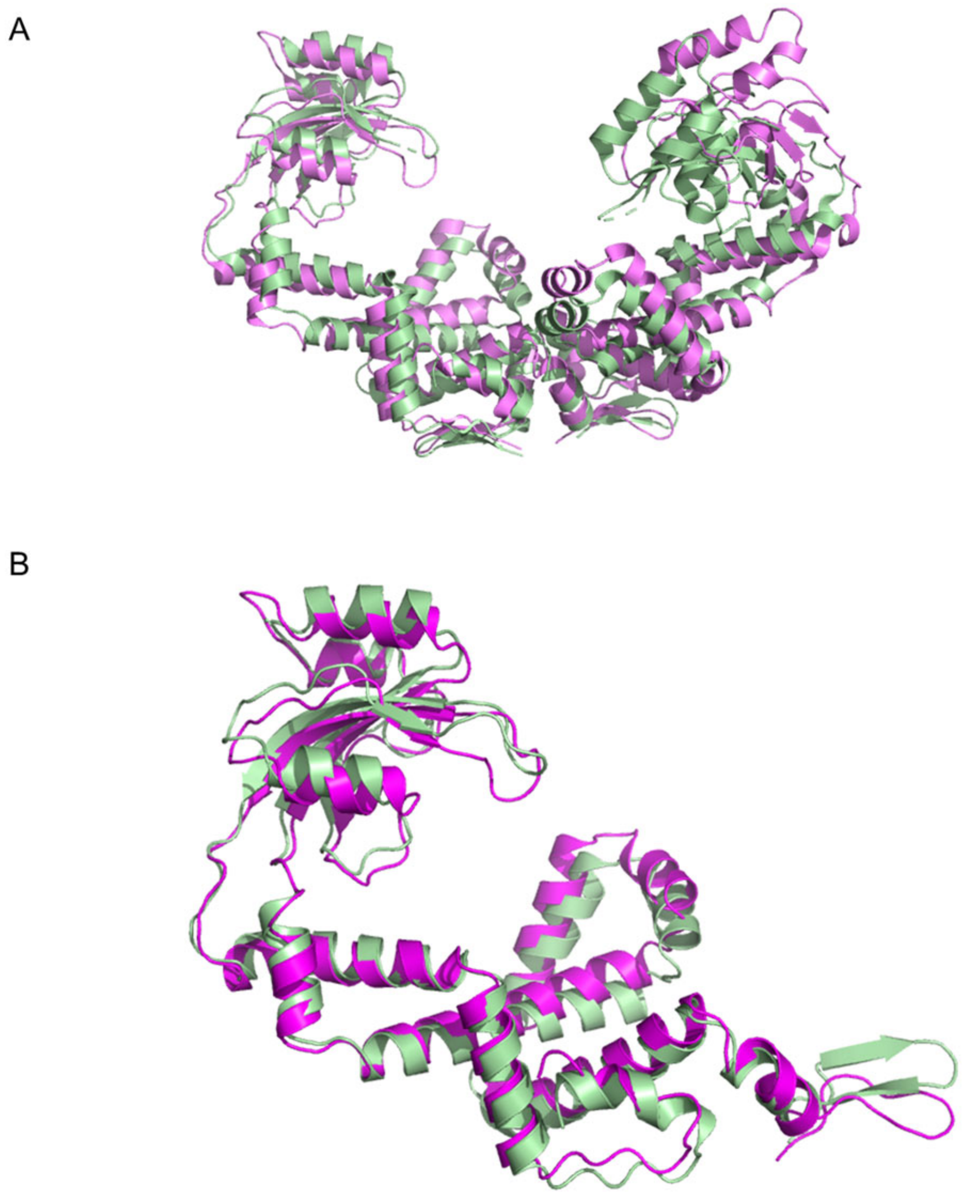

Figure S2: Structural comparison of *S. pneumoniae* PrsA and *S. mutans* PrsA dimers. A) Cartoon representation of the alignment between structures of *Spn*PrsA (PDB 5TVL, Dimer 2, chain B & C, pale green) and *Smu*PrsA (PDB 7L75, Dimer 1, chain A & B, magenta) with a r.m.s.d. of 3.74 Å performed with PyMOL (2). B) A flexible alignment (3) of chain A from *Spn*PrsA (pale green) and *Smu*PrsA (violet) across 264 Cα pairs sharing 49 % sequence identity.

Figure S3

A

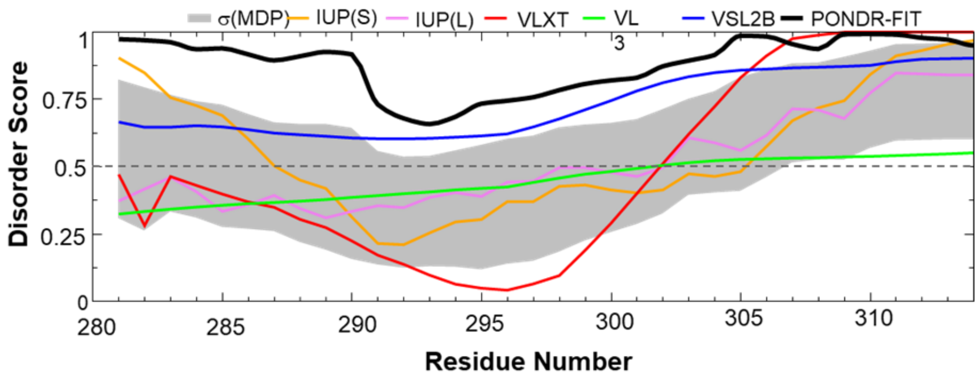

B

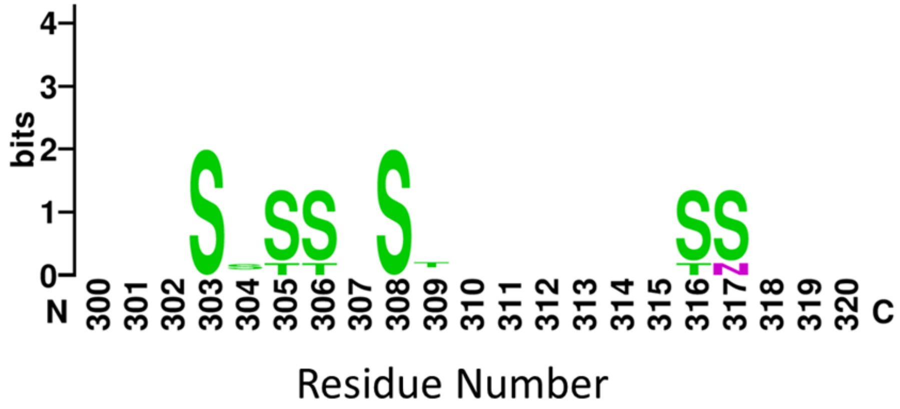

Figure S3. The C-terminal region of PrsA proteins is intrinsically disordered and enriched with serine residues. A. Predicted intrinsic disorder of PrsA amino acid residues 280-313. This prediction was made using the Rapid Intrinsic Disorder Analysis Online (RIDAO) v12.05.2021 tool. RIDAO generates six distinct intrinsic disorder prediction models including IUP (L OR S), VLXT, VL, VSL2B and PONDR-FIT. Disorder scores greater than the mean disorder profile (MDP) of 0.5 suggests a high degree of intrinsic disorder where amino acid positions with a score greater than 0.5 are disordered. The gray scale shows the error of the MDP, and the dotted line is the threshold between order and disorder. B.) Weblogo (4) image of the C-terminal region of PrsA proteins from *S. pneumoniae* (*Spn*PrsA; PDB 5TVL), *S. mutans* (*Smu*PrsA; PDB 7L75), *S. pyogenes* PrsA1 (*Spy*PrsA1; WP_011184661.1) and PrsA2 (*Spy*PrsA2; WP_010922719.1), *B. subtilis* (*Bsu*PrsA; PDB 4WO7), and *L. monocytogenes* PrsA1 (*Lmo*PrsA1; PDB 5HTF) and PrsA2 (*Lmo*PrsA2; WP_003724025.1). N and C indicate the N-terminal and C-terminal, respectively.

Figure S4

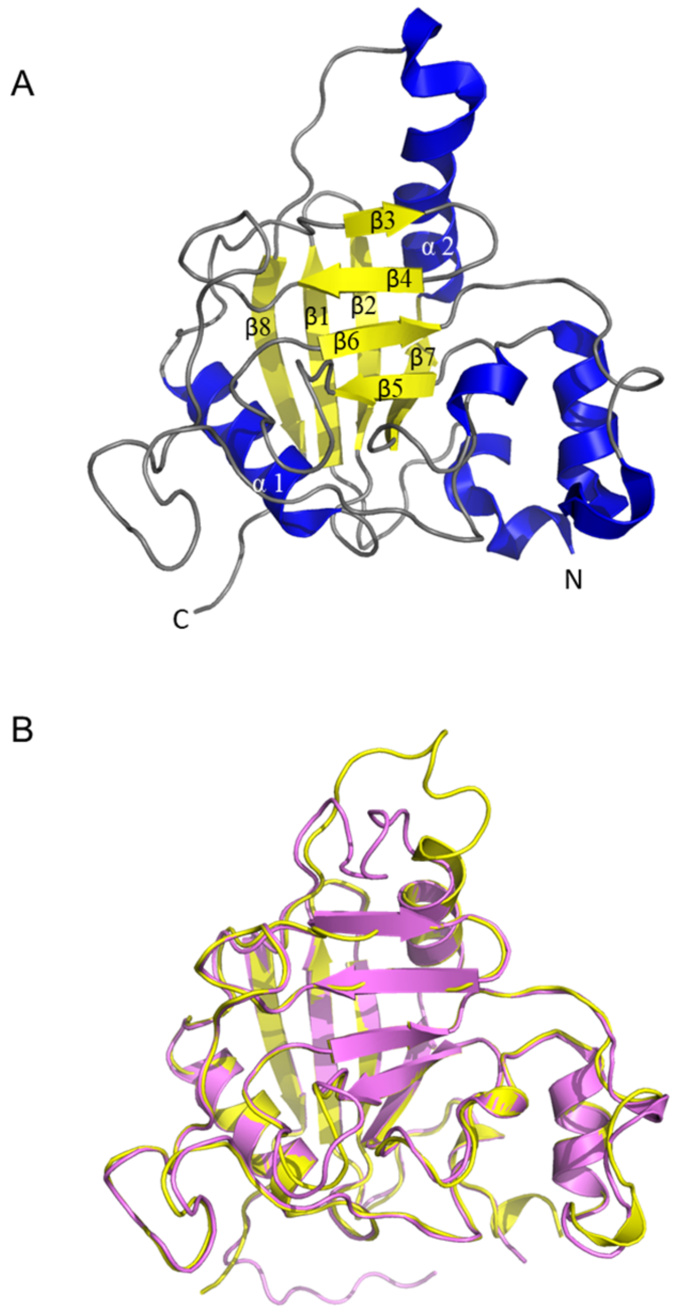

Figure S4: Structural comparison *S. pneumoniae* SlrA and *S. pyogenes* PpiA PPIase structures. A) The overall structure of *Spy*PpiA (PDB 7L6Y), depicted as a cartoon with helices in blue, β-strands in yellow and loop regions in grey. B) Superimposition of the structure of *Spn*SlrA (PDB 7L6Z, chain A, violet) and *Spy*PpiA (PDB 7L6Y, chain A, yellow). The two structures aligned with a r.m.s.d. of 2.08 Å across all carbon atoms. Alignment was done with PyMOL (2).

Figure S5

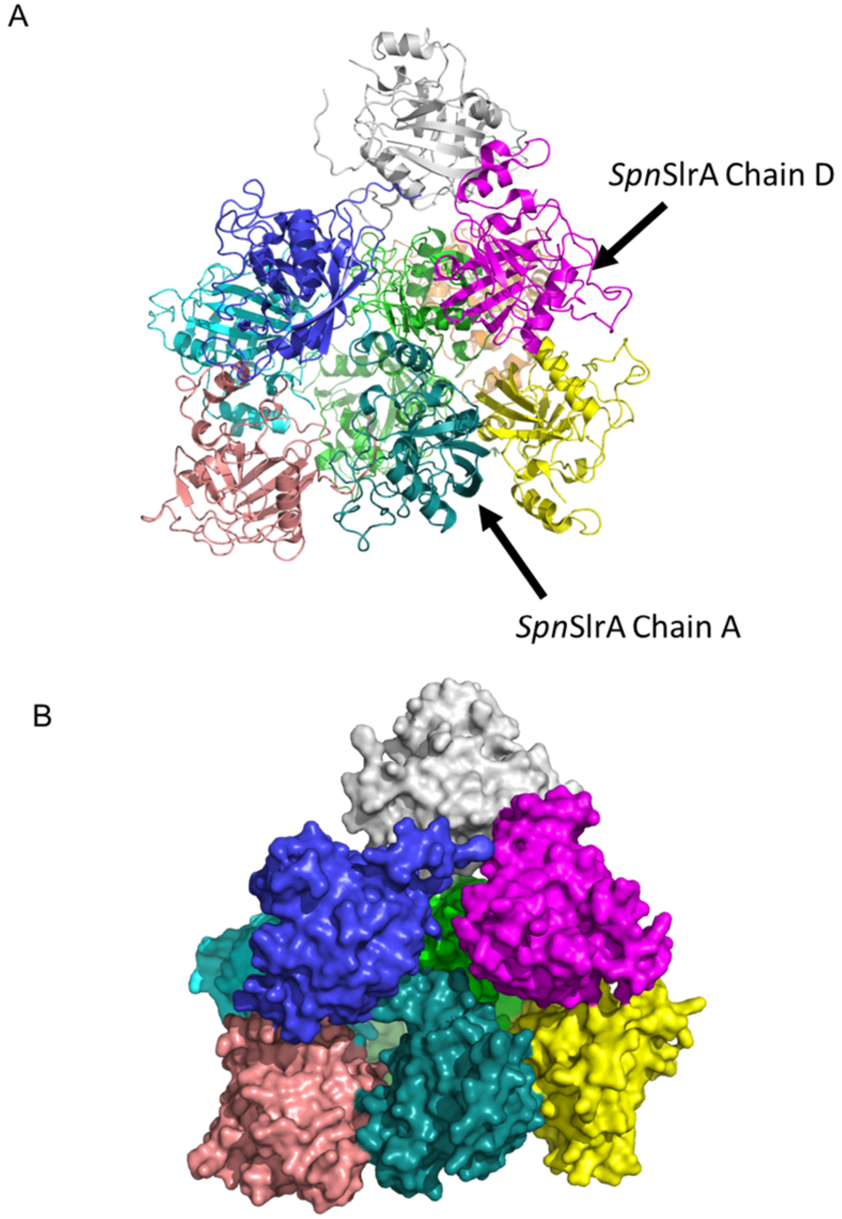

Figure S5: The asymmetric unit content of *S. pneumoniae* SlrA (PDB 7L6Z). The cartoon (A) and (B) surface representations of the asymmetric unit containing the 10 polypeptide chains of *Spn*SlrA (PDB 7L6Z), Chains A (deep teal), B (green), C (cyan), D (magenta), E (yellow), F (salmon), G (gray), H (blue), I (orange), J (lime). Chains A and D shown with arrows contain all residues in the recombinant protein (residues 61-267) in addition to a C-terminal 6-His-tag for purification. Structural models were visualized using PyMOL (2).

Figure S6

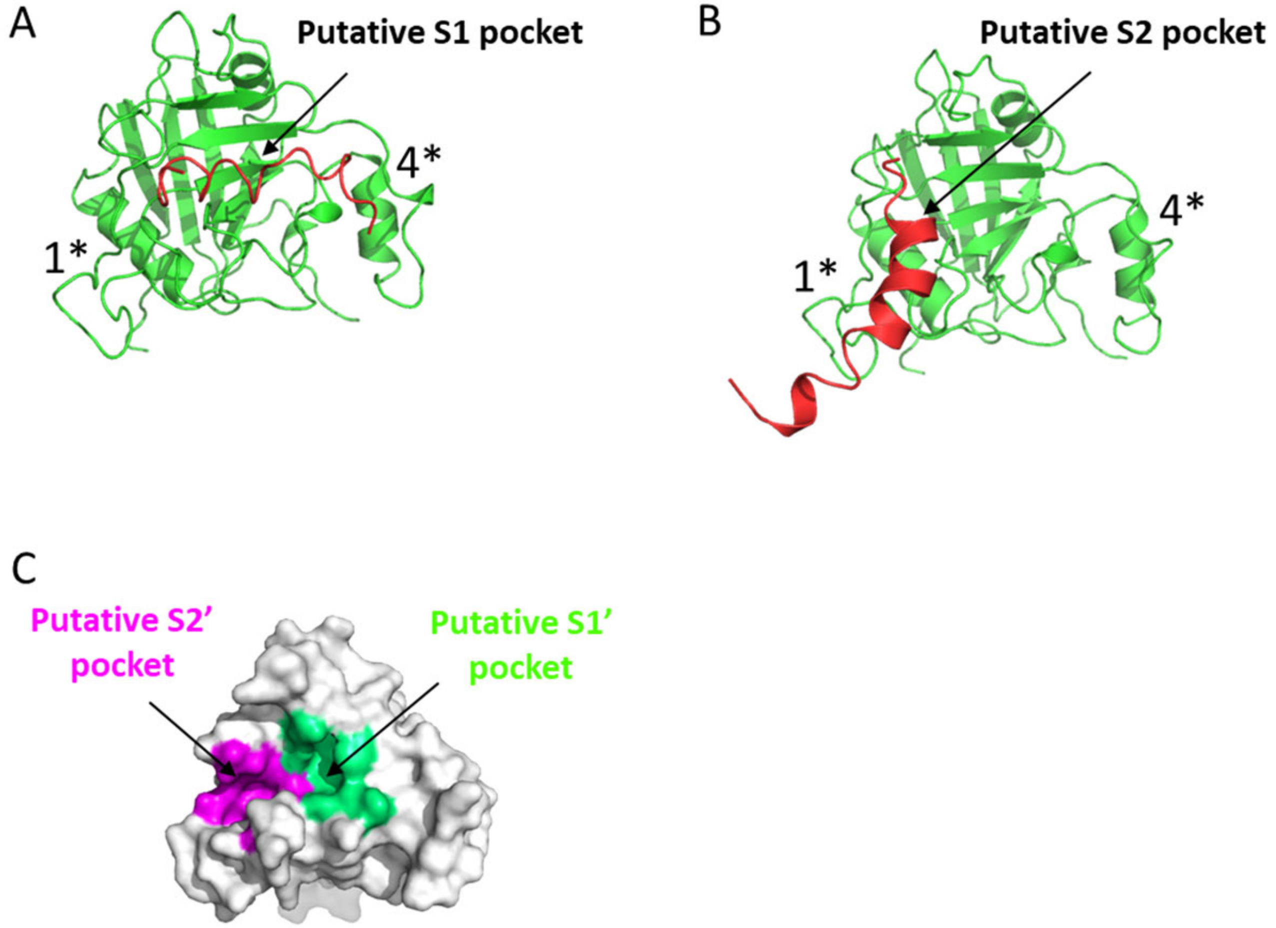

Figure S6. SlrA-like appendages may function as domains for substrate recognition or activity. A&B.) AlphaFold 3 (AF3) predicted multimer of *Spn*SlrA (PDB: 7l6Z, green) and 25 repeating string of the tetrapeptide AAPF (tv red). The two unique insertions/appendages are labelled as 1* and 4* respectively. Two conformations are shown with predicted interactions of the tetrapeptide with appendages 1 and 4 as well as the putative S1′ and S2′ pockets residues. C.) Putative β-barrel-like active site of SlrA colored as magenta and green shown in the SlrA cartoon structure.

Figure S7

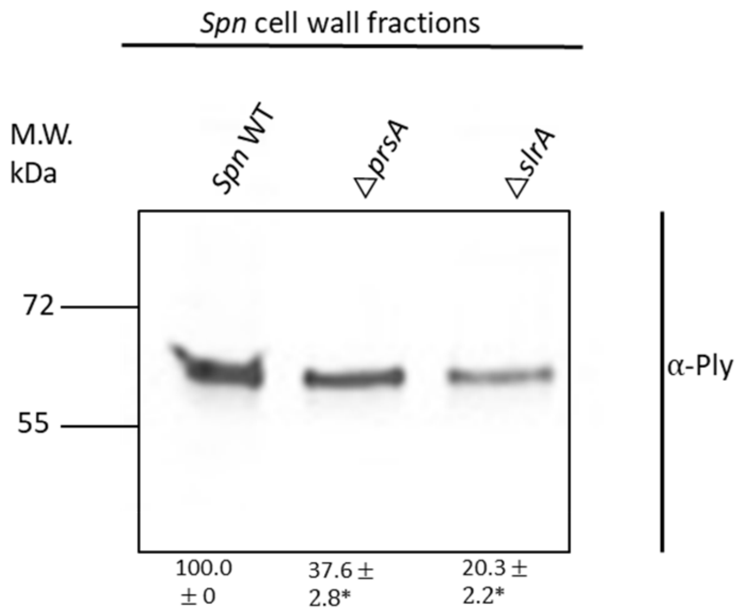

Figure S7. Pneumolysin is significantly reduced in the cell wall fraction of ***prsA*** **and** ***slrA*** **deletion mutants.** Immunoblot of cell wall fractions from *Spn* WT, △*prsA* and △*slrA* strains probed for pneumolysin (Ply) toxin levels with an antibody (α) directed toward Ply. Densitometry from three independent blots from three independent mutants was performed using ImageJ. One-way ANOVA with Tukey pairwise comparison test was used to compare the percent differences of △*prsA* and △*slrA* strains to the wildtype strain set at 100 percent where $*P<0.01$.

Figure S8

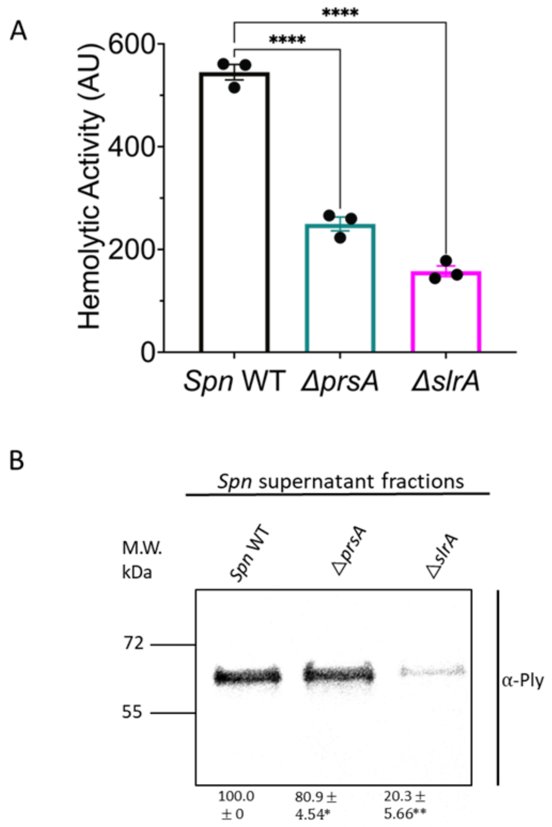

Figure S8. PrsA and SlrA are required for the secretion of functional pneumolysin. A.) Hemolytic activity of released pneumolysin in supernatant fractions of *S. pneumoniae* wildtype (*Spn* WT), Δ*prsA* and Δ*slrA* strains. Hemoglobin released from sheep's red blood cell lysis was quantified in the form of hemolytic units using a plate reader. Hemolytic activity of the Δ*prsA* and Δ*slrA* strains were compared to the wildtype strain using one-way ANOVA with Dunnett's multiple comparisons test where **P*<0.0001. Data represent three independent experiments from three independent mutants. B.) Immunoblot of cell supernatants from *Spn* WT, △*prsA* and △*slrA* strains probed for the levels of the pneumolysin (Ply) toxin with an antibody (α) directed toward Ply. Densitometry from two independent blots from two independent mutants was performed using ImageJ. One-way ANOVA with Tukey pairwise comparison was used to compare the percent differences of △*prsA* and △*slrA* strains to the wildtype strain set at 100 percent where **P*<0.05 and ***P*<0.01.

Figure S9

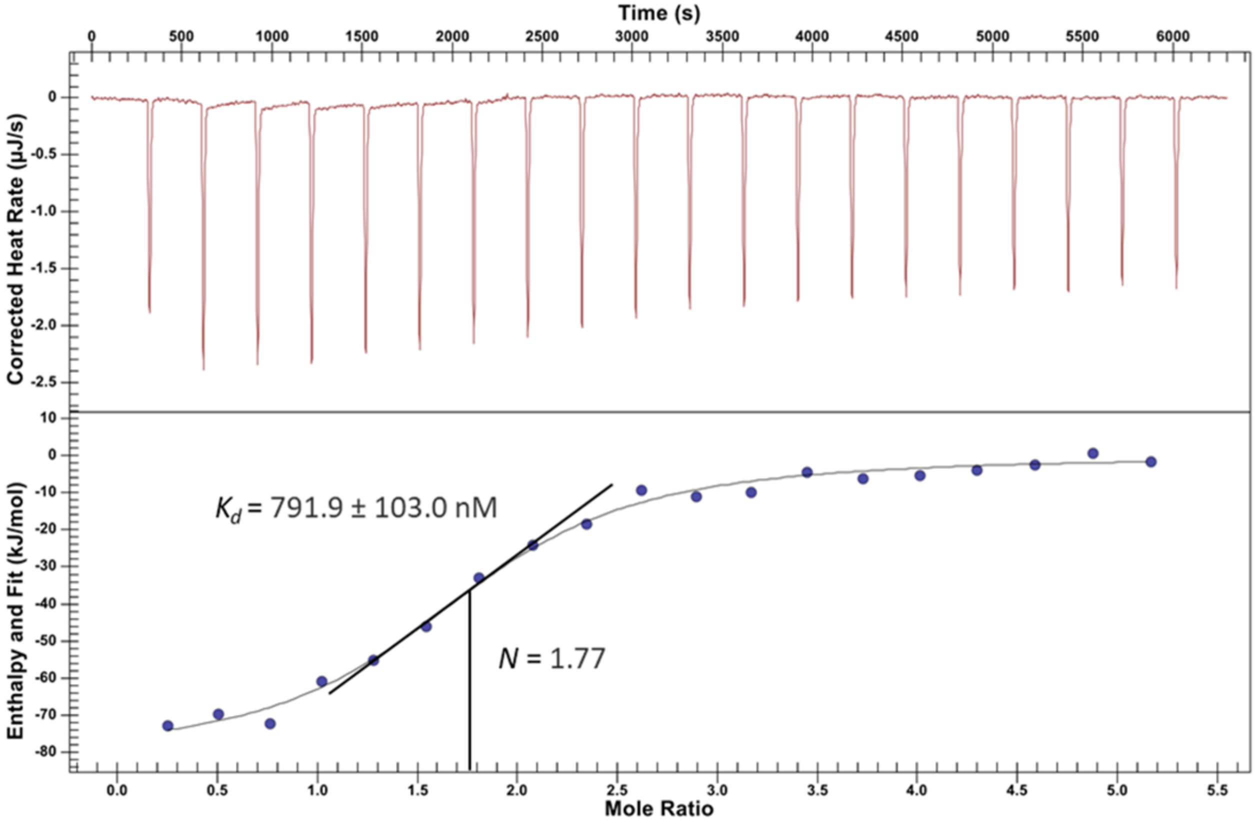

Figure S9. The PPIase domain of *S. pneumoniae* PrsA is required for efficient binding to the pneumolysin, Ply. Binding interaction between *Spn*PrsA N+C and Ply was determined using affinity isothermal titration calorimetry (ITC). PrsA N+C (78 µM) in the syringe was titrated against Ply (2.6 µM) in the sample cell. The solid line corresponds to the fitted curve with a molar ratio of approximately 2:1 (*N* is 1.77), the K_d is 791.9 ± 103.0 nM, ΔH is -81.50 ± 4.553 kJ/mol, K_a is 6.67 x10^6 M^{-1}, ΔG is -36.69 kJ/mol, and ΔS is -150.3 J/mol·K. ITC thermograms are representative graphs of three independent experiments.

Figure S10

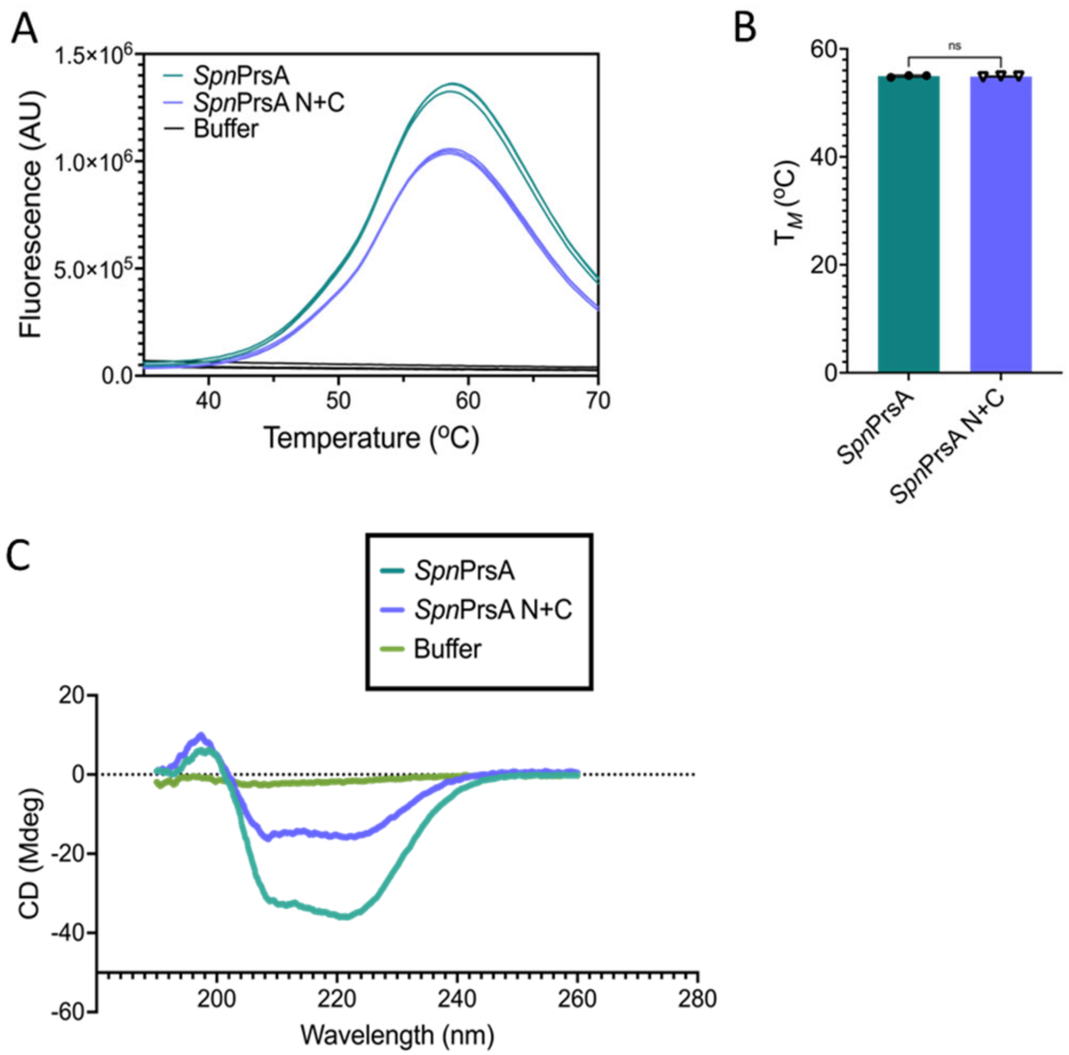

Figure S10. Thermal stability and circular dichroism spectroscopy (CD) analysis of *Spn*PrsA and PrsA N+C proteins. A) Thermal protein unfolding of *Spn*PrsA and *Spn*PrsA N+C along a temperature gradient. B) Melting temperatures (T_m) of recombinant *Spn*PrsA and *Spn*PrsA N+C were similar when compared by unpaired two-tailed Student's *t*-test. NS indicates not significant. C) CD spectroscopy analysis of the *Spn*PrsA and *Spn*PrsA N+C structures. Recombinant proteins were diluted using 20 mM KH_2PO_4, pH 7.4 buffer and CD spectra was collected at 25°C.

Figure S11

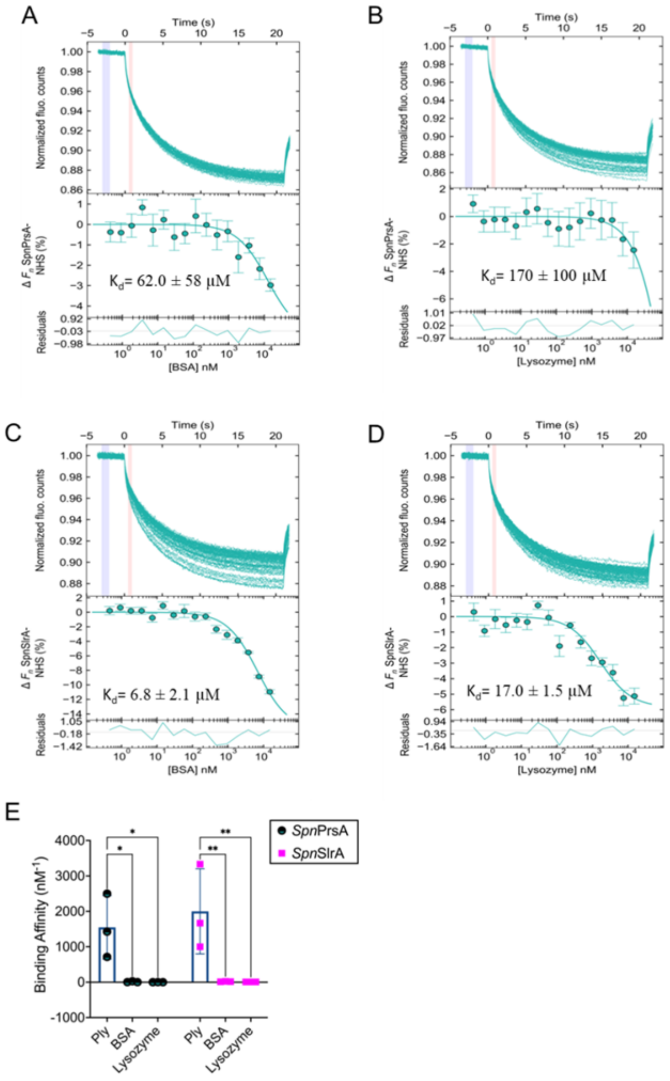

Figure S11. PrsA and SlrA exhibit poor affinity to bovine serum albumin (BSA) and lysozyme. A-D.) Microscale thermogram of *Spn*PrsA and SlrA titrated against the recombinant pneumolysin. The concentration of the labeled *Spn*PrsA or *Spn*SlrA was kept constant (20 nM) while a range of Ply (14 nM to 18000 nM) was used. Results were analyzed with PALMIST (5) and figures were plotted using GUSSI v1.2.0 (6). The dissociation constant (K_d) is 62.0 ± 58 μM and 170 ± 100 μM[ŧ] for PrsA bound to BSA and lysozyme, respectively. The K_d of SlrA bound to BSA and lysozyme is 6.8 ± 2.1 μM and 17.0 ± 1.5 μM[ŧ], respectively. The top panel shows the thermophoretic time-traces of three or more experiments and the blue and pink areas represent time spans used to obtain the fluorescence cold (F_c) and hot (F_h) regions, respectively. The middle panel shows the binding curve with the line of best fit using the 1:1 binding model (95% confidence interval), and the error bars represent the standard deviation. The residuals between the data and fit are shown in the bottom panel. ([ŧ]Thermophoretic fitting parameters are unstable). E) Comparison of the estimated binding affinity of PrsA or SlrA for Ply, BSA or lysozyme. Binding affinity was determined as 1/K_d. Binding affinity of PrsA or SlrA for Ply was compared with BSA and lysozyme using two-way ANOVA with Šídák's multiple comparisons test, *P<0.01 and **P<0.001. Data is represented as mean ± standard deviations of three independent experiments.

Figure S12

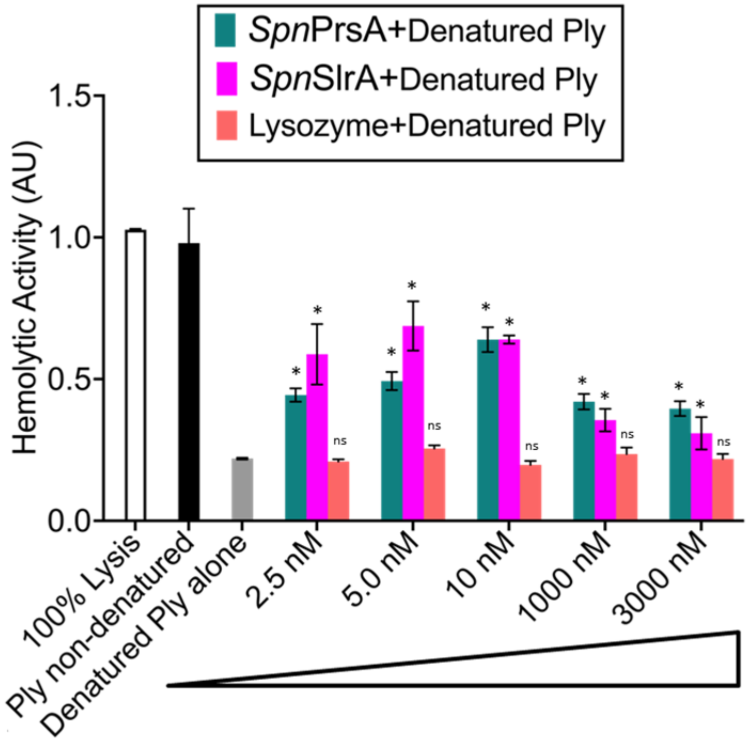

Figure S12. *S. pneumoniae* PrsA and SlrA demonstrate inhibitory protein folding or holdase activity at high concentrations. Chaperone-assisted folding assay of denatured Ply toxin. Recombinant Ply was denatured in a urea buffer and refolding of Ply (10 nM) was initiated in the presence of increasing concentrations of PrsA, SlrA, or the control protein lysozyme. Hemolytic activity in the presence of the chaperones and lysozyme was compared to the denatured Ply-alone (10 nM) using one-way ANOVA with Dunnett's multiple comparisons test, *P<0.01. Data represent three independent experiments and not significant (ns) differences are indicated.

Figure S13

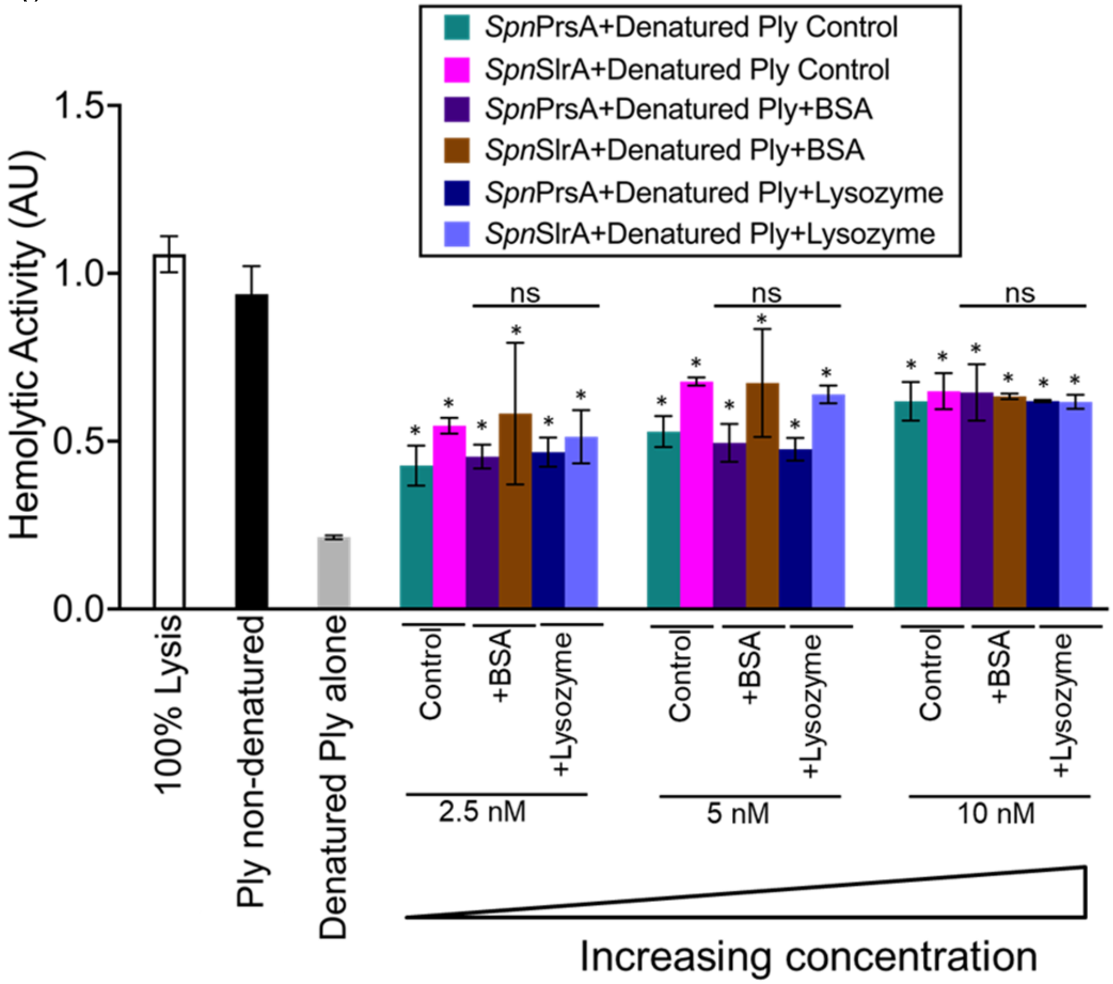

Figure S13. Specificity of *S. pneumoniae* PrsA and SlrA chaperones. Chaperone-assisted protein folding assay of denatured Ply. Recombinant Ply was denatured in a urea buffer and folding of Ply (10 nM) was initiated in the presence of increasing concentrations of PrsA or SlrA in addition to either serum albumin (BSA) or lysozyme. Hemolytic activity in the presence of BSA and lysozyme was compared to either PrsA or SlrA with denatured-Ply alone (10 nM) control using one-way ANOVA with Dunnett's multiple comparisons test. Differences between the data and the denatured Ply alone control were compared using one-way ANOVA with Dunnett's multiple comparisons test, *$P<0.01$. Data represents three independent experiments and differences that are not significant (ns) are indicated.

Figure S14

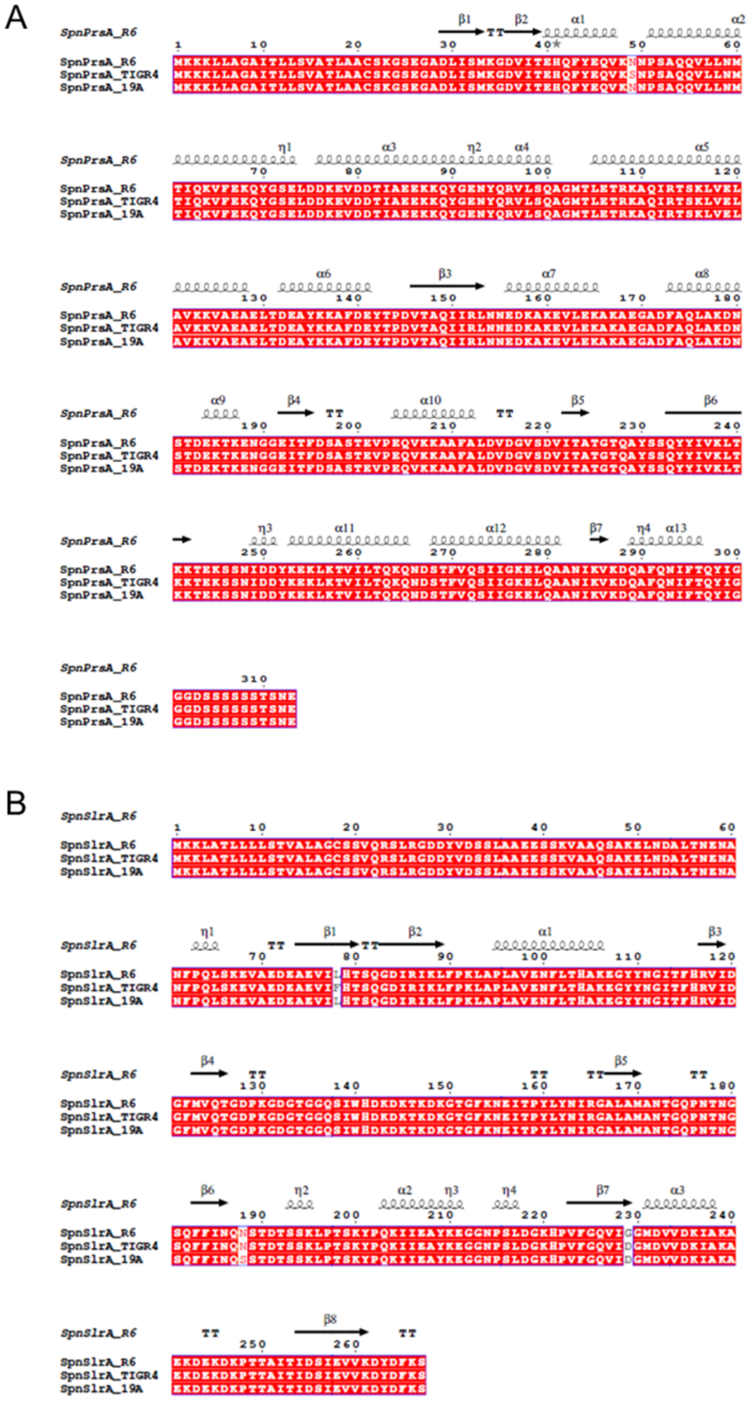

Figure S14. Conservation of PrsA and SlrA in *S. pneumoniae* strains used in this study. Primary amino acid sequence alignment of PrsA (A) and SlrA (B) in *S. pneumoniae* strains R6, TIGR4 and 19A. Sequence alignment was done using PROMALS3D (7) and the generated figure was rendered using ESPRIPT (8)**.**

Figure S15

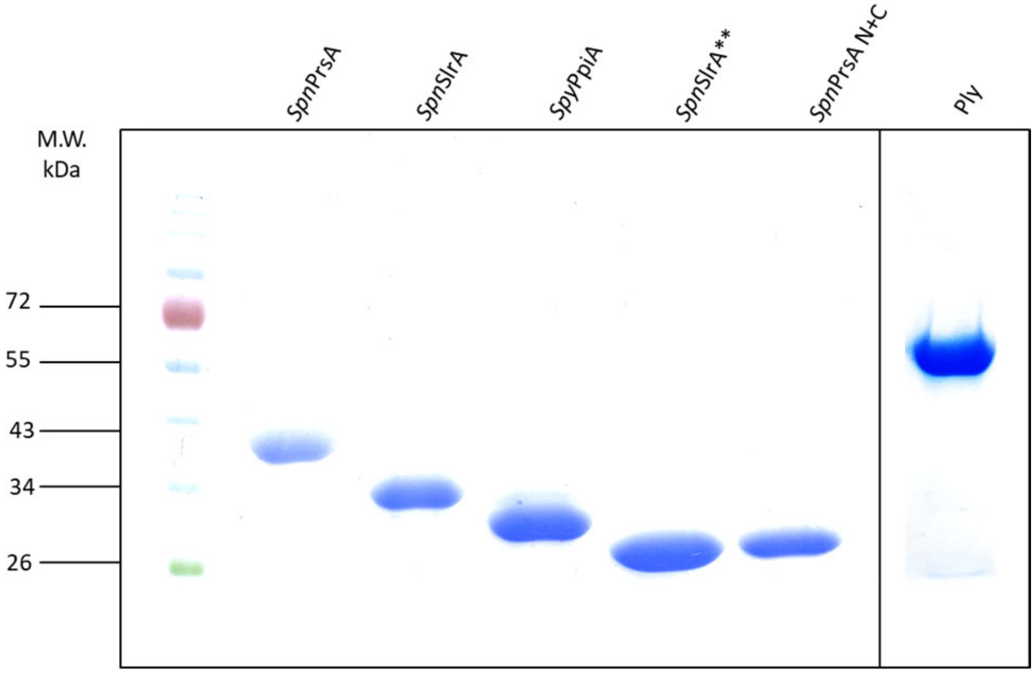

Figure S15. SDS-PAGE of purified recombinant proteins. Recombinant proteins were run using a 10 % SDS-PAGE gel at 200 V for 1 hour and stained with Coomassie stain R250 dye. Protein gel was de-stained overnight and imaged. Double asterisks (**) indicates the X-ray crystallized protein.

Table S1: List of bacterial strains and plasmids.

Strain/Plasmid	Genotype	Designation	Source/reference
TOP10	*E. coli* host strain used for Cloning		Invitrogen
LAC-153	*E. coli* BL21 (DE3Star) cells for protein expression and purification	DE3Star	Invitrogen
LAC-4438	*S. pneumoniae* TIGR4 Δ*cps*::*cam* #1, 2 and 3	*Spn* WT	(9)
LAC-4417<br>LAC-4418<br>LAC-4419	*S. pneumoniae* TIGR4 Δ*cps*::*cam*, Δ*prsA*::*tet* #1, 2 and 3	Δ*prsA*	(9)
LAC-4420<br>LAC-4421<br>LAC-4422	*S. pneumoniae* TIGR4 Δ*cps*::*cam*, Δ*slrA*::*spc* #1, 2 and 3	Δ*slrA*	(9)
LAC-2394	*S. pneumoniae* Δ*ply::spc*	Δ*ply*	(10)
LAC-4037	*S. pneumoniae* Δ*ply::spc + ply*	Δ*ply complement*	(10)
LAC-160	N-terminal protein expression vector	pQE30	QIAGEN
LAC-161	N-terminal protein expression vector	pMCSG53	CSBID*
LAC-162	C-terminal protein expression vector	pMSCG92	CSBID*
LAC-163	pMCSG53-*Spn*PrsA	*Spn*PrsA	This study
LAC-164	pMCSG53-*Smu*PrsA	*Smu*PrsA	This study
LAC-165	pMSCG92-*Spn*SlrA	*Spn*SlrA	This study
LAC-166	pMCSG53-*Spy*PpiA	*Spy*PpiA	This study
LAC-167	pMSCG92-*Spn*SlrA	*Spn*SlrA	This study
LAC-168	pQE30-Ply	Ply	This study
LAC-169	pQE30-*Spn*SlrA	*Spn*SlrA	This Study
LAC-170	pQE30-*Spn*PrsA N+C domain only	*Spn*PrsA N+C	This Study

*Center for Structural Biology of Infectious Diseases (CSBID), Northwestern, Feinberg School of Medicine.

Table S2: List of oligonucleotides.

Primer Name	Sequence (5’-3’)	Use
*Spn*PrsA_Fwd<br><br>*Spn*PrsA_Rev	TTGTATTTCCAGGGCGGGGCAGACCTTATCAGCATG<br><br>CAAGCTTCGTCATCATTCGTTTGATGTACTACTGCTTG	Cloning (Amino acid residues 27-313)
*Spn*PrsASacEX_Fwd<br><br>*Spn*PrsAXmaIEX_Rev	agtaGAGCTCTCGAAAGGGTCAGAAGGAG<br><br>agtaCCCGGGCTATTCGTTTGATGTACTAC	Cloning *Spn*PrsA N+C domain only
*Smu*PrsA_Fwd<br><br>*Smu*PrsA_Rev	TACTTCCAATCCAATGCCGCTTGTTCAAAAACAAAC CAAAACAGTAAAATTG<br><br>TTATCCACTTCCAATGTTAATATTGTGATAAGATCTC ACTAAAGGCCTTATCT	Cloning (Amino acid residues 21-294)
*Spn*SlrA_Fwd<br><br>*Spn*SlrA_Rev	AGGAGGTTAGATATGAATTTCCCACAACTATCTAAG GAAGTTGCT<br><br>TTGGAAGTAGAGGTTCTCAGATTTAAAATCGTAGTCT TTCACCACTTCGAT	Cloning (Amino acid residues 61-267)
*Spy*PpiA_Fwd<br><br>*Spy*PpiA_Rev	TACTTCCAATCCAATGCCGAATCTGTTGACCGCGCTA TAAAAGG<br><br>TTATCCACTTCCAATGTTAGTTTTTAAAGCGATAATCT TTGACAATGTCAATTGA	Cloning (Amino acid residues 21-268)
pQE30_Fwd<br><br>pQE30_Rev	CCCGAAAAGTGCCACCTG<br><br>CAGGTGGCACTTTTCGGG	Sanger sequencing
*Spn*SlrA_SacEx_Fwd<br><br>*Spn*SlrA_XmaEx_Rev	agtaGAGCTCAGCAGCGTCCAACGCAGT<br><br>agtaCCCGGGTTAAGATTTAAAATCGTAGTC	Cloning (Amino acid residues 19-267)
*Spn*SlrA_seq_Fwd	GGACGGTACAGGTGGTCAGT	Sanger sequencing
*Spn*Ply_SacEx_Fwd<br><br>*Spn*Ply_Xma	agtaGAGCTCCAAATAAAGCAGTAAATGAC<br><br>agtaCCCGGGCTAGTCATTTTCTACCTTATC	Cloning (Amino acids residues 2-471)

Supplemental Material References:

1. Krissinel E, Henrick K. 2007. Inference of macromolecular assemblies from crystalline state. Journal of molecular biology 372:774-797.
2. DeLano WL. 2002. The PyMOL molecular graphics system. http://www pymol org.
3. Li Z, Jaroszewski L, Iyer M, Sedova M, Godzik A. 2020. FATCAT 2.0: towards a better understanding of the structural diversity of proteins. Nucleic acids research 48:W60-W64.
4. Crooks GE, Hon G, Chandonia JM, Brenner SE. 2004. WebLogo: a sequence logo generator. Genome Res 14:1188-90.
5. Scheuermann TH, Padrick SB, Gardner KH, Brautigam CA. 2016. On the acquisition and analysis of microscale thermophoresis data. Analytical Biochemistry 496:79-93.
6. Brautigam CA. 2015. Calculations and publication-quality illustrations for analytical ultracentrifugation data, p 109-133, Methods in enzymology, vol 562. Elsevier.
7. Robert X, Gouet P. 2014. Deciphering key features in protein structures with the new ENDscript server. Nucleic Acids Research 42:W320-W324.
8. Sievers F, Wilm A, Dineen D, Gibson TJ, Karplus K, Li W, Lopez R, McWilliam H, Remmert M, Söding J. 2011. Fast, scalable generation of high-quality protein multiple sequence alignments using Clustal Omega. Molecular systems biology 7:539.
9. George JL, Agbavor C, Cabo LF, Cahoon LA. 2024. Streptococcus pneumoniae secretion chaperones PrsA, SlrA, and HtrA are required for competence, antibiotic resistance, colonization, and invasive disease. Infect Immun 92:e0049023.
10. Price KE, Greene NG, Camilli A. 2012. Export requirements of pneumolysin in Streptococcus pneumoniae. Journal of Bacteriology 194:3651-3660.
